# Metabolic vulnerability index and Life’s Essential 8 with risk of major adverse cardiovascular events

**DOI:** 10.1038/s44325-026-00139-0

**Published:** 2026-06-12

**Authors:** Xiaojie Wang, Yanhui Gao, Gregory Y. H. Lip, Jianwen Guo, Junguo Zhang

**Affiliations:** 1 Research Team of Prevention and Treatment of Cerebral Hemorrhage Applying Chinese Medicine, The Second Affiliated Hospital of Guangzhou University of Chinese Medicine, Guangzhou University of Chinese Medicine, Guangzhou, China; 2 Department of Medical Statistics, School of Basic Medicine and Public Health, Jinan University, Guangzhou, China; 3 Liverpool Centre for Cardiovascular Science at University of Liverpool, Liverpool John Moores University and Liverpool Heart & Chest Hospital, Liverpool, UK; 4 Danish Center for Health Services Research, Department of Clinical Medicine, Aalborg University, Aalborg, Denmark; 5 Department of Cardiology, Lipidology and Internal Medicine with Intensive Coronary Care Unit, Medical University of Bialystok, Bialystok, Poland; 6 State Key Laboratory of Traditional Chinese Medicine Syndrome, The Second Affiliated Hospital of Guangzhou University of Chinese Medicine, Guangzhou, China; 7 Guangdong Provincial Key Laboratory of Research on Emergency in Traditional Chinese Medicine, Guangzhou, China; 8 School of Basic Medical Sciences, Guangzhou University of Chinese Medicine, Guangzhou, China

**Keywords:** Biomarkers, Cardiology, Diseases, Medical research, Risk factors

## Abstract

The metabolic vulnerability index (MVX) captures metabolic-inflammatory vulnerability, but its joint relevance with Life’s Essential 8 (LE8) for major adverse cardiovascular events (MACE) is unclear. We analyzed 239,135 UK Biobank participants free of baseline MACE. MVX was calculated from six NMR-based biomarkers. LE8 scores were classified as low (<60), moderate (60–79), or high (≥80) cardiovascular health (CVH). Cox models evaluated associations of MVX and LE8 with incident MACE; joint-effect, interaction, counterfactual, and mediation analyses were conducted. Over a median 13.6 years, 17,146 MACE occurred. Higher MVX was associated with higher MACE risk (HR = 1.08, 95% CI: 1.07, 1.10), whereas high CVH was associated with lower risk compared to low CVH (HR = 0.44, 95% CI: 0.41, 0.47). Participants with low CVH and MVX Q4 had the highest risk (HR = 2.84, 95% CI: 2.60, 3.12). Additive interaction was evident for MACE and myocardial infarction. Counterfactual and mediation analyses suggested that better CVH could prevent a substantial proportion of events, partly through lower metabolic vulnerability. Combined MVX and LE8 assessment may improve cardiovascular risk stratification and support targeted prevention.

## Introduction

Metabolic dysregulation and systemic inflammation are established contributors to the pathogenesis of cardiovascular disease (CVD)^1,2^. The metabolic vulnerability index (MVX), derived from nuclear magnetic resonance (NMR) metabolomics, integrates biomarkers of inflammation and metabolic stress, including glycoprotein acetyls (GlycA), amino acid metabolites, and lipid particles^3^. Elevated MVX has been shown to predict all-cause mortality with prognostic power exceeding established risk factors, even in apparently healthy individuals^4^. Unlike conventional inflammatory biomarkers such as C-reactive protein (CRP) or interleukin-6 (IL-6), which mainly reflect specific inflammatory activity, MVX provides a more integrated metabolomics-based profile of metabolic-inflammatory vulnerability. Compared with broader metabolomics-based prediction models, MVX is a relatively parsimonious composite index with greater biological interpretability^5,6^. Mechanistically, high MVX reflects a milieu of chronic inflammation, oxidative stress, protein-energy imbalance, and impaired metabolic resilience that accelerates atherogenesis and promotes plaque instability, predisposing to major adverse cardiovascular events (MACE).

While intrinsic metabolic vulnerability may be only partially modifiable, behavioral and clinical factors substantially influence both inflammation and metabolic homeostasis. The American Heart Association’s Life’s Essential 8 (LE8) provides a comprehensive measure of cardiovascular health (CVH), encompassing diet, physical activity, smoking, sleep, BMI, blood pressure, blood glucose, and non-HDL cholesterol^7^. High LE8 scores consistently predict reduced cardiovascular events and mortality^8,9^. Notably, LE8 components directly modulate the inflammatory-metabolic pathways induced by MVX, suggesting potential risk mitigation through lifestyle optimization^10^.

However, prior research has predominantly focused on MVX and LE8 in isolation. MVX is well validated as a marker of intrinsic metabolic risk, whereas LE8 reflects modifiable behavioral and clinical determinants of CVH^1,11^. Evidence regarding their joint associations and potential interactions remains limited. This isolated approach limits our understanding of precision prevention, particularly whether the risk associated with high MVX is fixed or can be attenuated through CVH optimization.

Conceptually, MVX and LE8 capture two distinct but complementary dimensions of cardiovascular risk. MVX reflects underlying biological susceptibility that may precede overt disease, whereas LE8 represents modifiable health factors that are potentially amenable to intervention. Joint evaluation of these measures may therefore provide a more comprehensive framework for identifying high-risk individuals and estimating the extent to which optimal CVH may offset metabolically driven cardiovascular risk.

Hence, in this analysis from UK Biobank, we aimed to: (1) assess the independent and combined associations of MVX and LE8 with MACE risk, (2) determine whether favorable LE8 modifies the MVX–MACE association, and (3) use counterfactual analysis to estimate the potential reduction in MACE cases from improved LE8.

## Results

The baseline characteristics of the final included participants are shown in Table 1. Among the 239,135 participants, the mean age was 55.97 ± 8.11 years, 52.23% were female, and 95.33% were White. The raw MVX score was 27.08 ± 2.85 (range, 23.82–34.94), and the total LE8 score was 69.95 ± 11.53 (range, 13.13–100.00). Overall, 20.34% of participants had high CVH, 61.17% had moderate CVH, and 18.49% had low CVH.

**Table 1:** Baseline characteristics of 239,135 participants included in this analysis

Characteristics	Total	No incident MACE	Incident MACE	*P*-value
**Number of participants**	239,135	221,989	17,146	
**Age, mean** ± **SD, years**	55.97 ± 8.11	55.63 ± 8.09	60.36 ± 6.98	<0.001
**Sex**				<0.001
Female	124,897 (52.23)	118,927 (53.57)	5970 (34.82)	
Male	114,238 (47.77)	103,062 (46.43)	11,176 (65.18)	
**Ethnicity**				<0.001
White	227,977 (95.33)	211,535 (95.29)	16,442 (95.89)	
Non-White	11,158 (4.67)	10,454 (4.71)	704 (4.11)	
**Townsend deprivation index, mean** ± **SD**	−1.48 ± 2.98	−1.50 ± 2.96	−1.14 ± 3.16	<0.001
**Binge drinking**				<0.001
No	232,156 (97.08)	215,760 (97.19)	16,396 (95.63)	
Yes	6979 (2.92)	6229 (2.81)	750 (4.37)	
**Raw MVX score, mean** ± **SD (range)**	27.08 ± 2.85 (23.82–34.94)	27.00 ± 2.84 (23.82–34.94)	28.13 ± 2.79 (24.05–34.93)	<0.001
**GlycA, μmol/L, mean** ± **SD**	813.00 ± 119.44	810.77 ± 118.85	841.92 ± 123.24	<0.001
**sHDL, μmol/L, mean** ± **SD**	9.87 ± 1.32	9.88 ± 1.32	9.78 ± 1.35	<0.001
**Citrate, μmol/L, mean** ± **SD**	66.78 ± 13.19	66.75 ± 13.18	67.09 ± 13.28	<0.001
**Isoleucine, μmol/L, mean** ± **SD**	52.06 ± 17.78	51.89 ± 17.74	54.30 ± 18.19	<0.001
**Leucine, μmol/L, mean** ± **SD**	105.96 ± 28.39	105.69 ± 28.31	109.39 ± 29.19	<0.001
**Valine, μmol/L, mean** ± **SD**	212.67 ± 43.10	212.20 ± 42.96	218.78 ± 44.36	<0.001
**AHA LE8 Scores (out of 100 possible points), mean** ± **SD**				
Mean Total CVH Score (range)	69.95 ± 11.53 (13.13–100.00)	70.37 ± 11.42 (13.13–100.00)	64.56 ± 11.62 (13.13–97.50)	<0.001
Dietary recommendations for cardiovascular health	62.13 ± 17.43	62.14 ± 17.39	61.99 ± 17.98	<0.001
Physical activity	77.94 ± 36.39	78.20 ± 36.21	74.63 ± 38.59	<0.001
Tobacco/nicotine exposure	76.61 ± 31.56	77.20 ± 31.18	68.94 ± 35.15	<0.001
Sleep health	89.72 ± 18.22	89.91 ± 18.01	87.24 ± 20.52	<0.001
Body mass index	70.23 ± 28.00	70.79 ± 27.82	63.03 ± 29.32	<0.001
Blood lipids (non-HDL cholesterol)	47.72 ± 28.97	47.89 ± 28.97	45.53 ± 28.93	<0.001
Blood glucose	91.88 ± 18.54	92.39 ± 17.90	85.31 ± 24.45	<0.001
Blood pressure	43.38 ± 32.36	44.43 ± 32.43	29.81 ± 28.22	<0.001
**LE8 score stratification**				<0.001
High CVH (≥80)	48,639 (20.34)	47,113 (21.22)	1526 (8.90)	
Moderate CVH (60–79)	146,283 (61.17)	136,166 (61.34)	10,117 (59.01)	
Low CVH (<60)	44,213 (18.49)	38,710 (17.44)	5503 (32.09)	
**Use of antihypertensive medications**				<0.001
No	193,317 (80.84)	182,495 (82.21)	10,822 (63.12)	
Yes	45,818 (19.16)	39,494 (17.79)	6324 (36.88)	
**Use of lipid-lowering medications**				<0.001
No	206,756 (86.46)	194,149 (87.46)	12,607 (73.53)	
Yes	32,379 (13.54)	27,840 (12.54)	4539 (26.47)	
**Use of antidiabetic medications**				<0.001
No	231,434 (96.78)	215,688 (97.16)	15,746 (91.83)	
Yes	7701 (3.22)	6301 (2.84)	1400 (8.17)	

*SD* standard deviation, *AHA* American Heart Association, *LE8* Life’s Essential 8, *CVH* cardiovascular health, *MVX* metabolic vulnerability index, *GlycA* glycoprotein acetyls, *sHDL* particle number of small high-density lipoprotein.

Compared with participants without MACE, those who developed MACE were older, more often male, and had a higher Townsend deprivation index. They also had a less favorable metabolic profile, with a higher raw MVX score, higher levels of GlycA, citrate, isoleucine, leucine, and valine, and lower sHDL levels. In addition, participants with MACE had a lower total LE8 score and poorer scores for several LE8 components, and were more frequently classified as having low CVH and less frequently as having high CVH. Use of antihypertensive, lipid-lowering, and antidiabetic medications was also more common among participants who developed MACE. Baseline characteristics of participants included in and excluded from the analyses are shown in Supplementary Table 1. Most measured characteristics were similar between the two groups, with absolute standardized mean differences below 0.1 for the majority of variables.

### Associations of MVX and LE8 with risk of MACE

Over a median follow-up of 13.6 (IQR: 12.8–14.3) years, a total of 17,146 incident MACE occurred among 239,135 participants. In separate outcome-specific analyses, there were 7301 cases of HF, 8390 MI, 6017 stroke, and 3225 CVD deaths.

As shown in Table 2, higher MVX was associated with increased risk of MACE. In Model 1, each 1-SD increase in MVX was associated with a higher risk of MACE (HR = 1.17, 95% CI: 1.16, 1.19), and participants in Q4 had a 50% higher risk than those in Q1 (HR = 1.50, 95% CI: 1.44, 1.57). After further adjustment in Model 3, the associations differed in magnitude across the individual endpoints, being strongest for CVD mortality and MI, intermediate for HF, and weakest for stroke. Per 1-SD increase in MVX, the HRs (95% CIs) were 1.12 (1.08, 1.16) for CVD mortality, 1.10 (1.08, 1.13) for MI, 1.07 (1.04, 1.09) for HF, and 1.04 (1.01, 1.06) for stroke. Similarly, comparing the highest with the lowest MVX quartile, the corresponding HRs (95% CIs) were 1.36 (1.23, 1.51), 1.32 (1.24, 1.41), 1.13 (1.05, 1.20), and 1.06 (0.98, 1.14), respectively. The adjusted population attributable fraction (PAF) for Q4 was 16.58% for MACE, 8.64% for stroke, 14.17% for HF, 22.48% for MI, and 24.97% for CVD mortality.

**Table 2:** Associations of MVX quartile with risk of MACE

Characteristics	Model 1^a^		Model 2^b^		Model 3^c^		PAF% (95% CI)
	HR (95% CI)	*P* value	HR (95% CI)	*P* value	HR (95% CI)	*P* value	
**MACE**							
per 1-SD increase	1.17 (1.16, 1.19)	<0.001	1.14 (1.12, 1.16)	<0.001	1.08 (1.07, 1.10)	<0.001	
Q1	-		-		-		
Q2	1.13 (1.08, 1.18)	<0.001	1.11 (1.06, 1.16)	<0.001	1.05 (1.00, 1.10)	0.039	4.75 (2.52, 6.94)
Q3	1.27 (1.22, 1.33)	<0.001	1.23 (1.17, 1.28)	<0.001	1.12 (1.07, 1.17)	<0.001	9.82 (7.63, 11.95)
Q4	1.50 (1.44, 1.57)	<0.001	1.40 (1.34, 1.46)	<0.001	1.21 (1.15, 1.26)	<0.001	16.58 (14.47, 18.63)
**Stroke**							
per 1-SD increase	1.10 (1.08, 1.13)	<0.001	1.08 (1.05, 1.11)	<0.001	1.04 (1.01, 1.06)	0.008	
Q1	-		-		-		
Q2	1.07 (0.99, 1.15)	0.091	1.05 (0.98, 1.14)	0.159	1.02 (0.94, 1.09)	0.676	2.71 (0.00, 6.34)
Q3	1.11 (1.03, 1.19)	0.008	1.08 (1.00, 1.16)	0.045	1.01 (0.94, 1.09)	0.827	3.68 (0.00, 7.33)
Q4	1.25 (1.16, 1.34)	<0.001	1.18 (1.10, 1.27)	<0.001	1.06 (0.98, 1.14)	0.124	8.64 (4.91, 12.22)
**HF**							
per 1-SD increase	1.18 (1.15, 1.20)	<0.001	1.13 (1.10, 1.15)	<0.001	1.07 (1.04, 1.09)	<0.001	
Q1	-		-		-		
Q2	1.07 (1.00, 1.15)	0.060	1.04 (0.97, 1.12)	0.222	0.99 (0.92, 1.06)	0.761	2.32 (0.00, 5.75)
Q3	1.20 (1.12, 1.29)	<0.001	1.14 (1.06, 1.22)	<0.001	1.03 (0.97, 1.11)	0.343	6.66 (3.14, 10.05)
Q4	1.46 (1.37, 1.56)	<0.001	1.32 (1.23, 1.41)	<0.001	1.13 (1.05, 1.20)	<0.001	14.17 (10.78, 17.44)
**MI**							
per 1-SD increase	1.20 (1.18, 1.23)	<0.001	1.17 (1.15, 1.20)	<0.001	1.10 (1.08, 1.13)	<0.001	
Q1	-		-		-		
Q2	1.22 (1.14, 1.30)	<0.001	1.19 (1.12, 1.28)	<0.001	1.12 (1.05, 1.19)	<0.001	8.34 (5.03, 11.53)
Q3	1.42 (1.33, 1.51)	<0.001	1.37 (1.28, 1.46)	<0.001	1.22 (1.15, 1.30)	<0.001	15.28 (12.10, 18.35)
Q4	1.69 (1.58, 1.79)	<0.001	1.58 (1.48, 1.68)	<0.001	1.32 (1.24, 1.41)	<0.001	22.48 (19.45, 25.39)
**CVD mortality**							
per 1-SD increase	1.24 (1.21, 1.28)	<0.001	1.20 (1.16, 1.24)	<0.001	1.12 (1.08, 1.16)	<0.001	
Q1	-		-		-		
Q2	1.22 (1.10, 1.36)	<0.001	1.20 (1.07, 1.33)	<0.001	1.12 (1.00, 1.25)	0.042	8.96 (3.36, 14.24)
Q3	1.41 (1.27, 1.56)	<0.001	1.33 (1.20, 1.48)	<0.001	1.19 (1.07, 1.32)	<0.001	14.64 (9.19, 19.78)
Q4	1.81 (1.64, 2.01)	<0.001	1.64 (1.48, 1.82)	<0.001	1.36 (1.23, 1.51)	<0.001	24.97 (19.91, 29.70)

*MACE* Major adverse cardiovascular events, *HF* heart failure, HR hazard ratio, *C* confidence interval; *PAF* population attributable fraction, *CVD* cardiovascular disease, *MI* myocardial infarction.

^a^Model 1 adjusted for age and sex.

^b^Model 2 additionally adjusted for ethnicity, Townsend deprivation index, binge drinking, baseline use of antihypertensive, lipid-lowering, and antidiabetic medications.

^c^Model 3 adjusted for terms in model 2 and LE8 category.

Conversely, higher LE8 was strongly associated with lower MACE risk (Supplementary Table 2). Each 1-point increase in LE8 was associated with a 3% lower risk of MACE (HR = 0.97, 95% CI: 0.97, 0.97). Compared with low CVH, high CVH was associated with lower risks of MACE (HR = 0.44, 95% CI: 0.41, 0.47). Across the individual components of MACE, the strength of association also varied, with the greatest relative risk reductions observed for CVD mortality (HR = 0.36, 95% CI: 0.31, 0.42) and MI (HR = 0.37, 95% CI: 0.34, 0.40), followed by HF (HR = 0.42, 95% CI: 0.38, 0.46) and stroke (HR = 0.55, 95% CI: 0.50, 0.61). Restricted cubic spline analyses showed overall significant associations between MVX and MACE, MI, HF, stroke, and CVD mortality. A significant non-linear association was observed only for MI (*P* for non-linearity=0.007), whereas the associations for MACE, HF, stroke, and CVD mortality were consistent with linearity (*P* for non-linearity=0.489, 0.108, 0.290, and 0.230, respectively; Supplementary Figs. 1–5).

### Joint associations of MVX and LE8 with risk of MACE

**Fig. 1:**
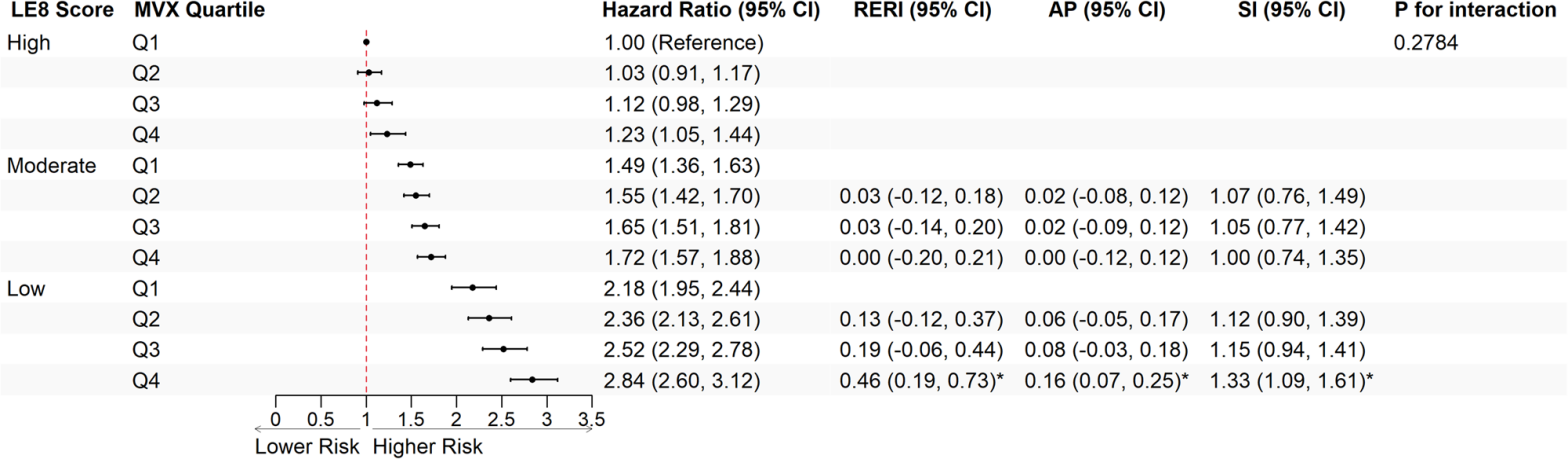
Combined effects of metabolic vulnerability index (MVX) quartile and Life’s Essential 8 (LE8) on the risk of major adverse cardiovascular events. The forest plot displays the Hazard Ratios (HRs) and 95% Confidence Intervals (CIs) across different combinations of LE8 scores and MVX quartiles, with the High LE8 and MVX Q1 group serving as the reference. Additive interaction metrics, including the Relative Excess Risk due to Interaction (RERI), Attributable Proportion (AP), and Synergy Index (SI), are also presented. Error bars indicate the 95% CIs.

Compared with participants with high CVH in the lowest MVX quartile, those with low CVH in the highest MVX quartile had the highest risk of MACE (HR = 2.84, 95% CI: 2.60, 3.12), stroke (HR = 1.87, 95% CI: 1.62, 2.15), HF (HR = 2.76, 95% CI: 2.39, 3.20), MI (HR = 3.79, 95% CI: 3.29, 4.36), and CVD mortality (HR = 3.71, 95% CI: 2.94, 4.68). Across all outcomes, risk increased progressively with worsening LE8 categories and higher MVX quartiles, with the lowest risks generally observed among participants with high CVH regardless of MVX quartile. For MACE and MI, additive interaction analyses showed evidence of positive interaction in the highest-risk category, whereas for stroke, HF, and CVD mortality, the joint associations were directionally similar but neither additive nor multiplicative interaction reached statistical significance (Fig. 1**;** Supplementary Figs. 6–9). Consistent with these findings, Supplementary Table 3 showed higher crude event proportions across worsening CVH categories and higher MVX quartiles, with the highest proportions observed in the low-CVH/MVX-Q4 group.

### Counterfactual analyses

**Fig. 2:**
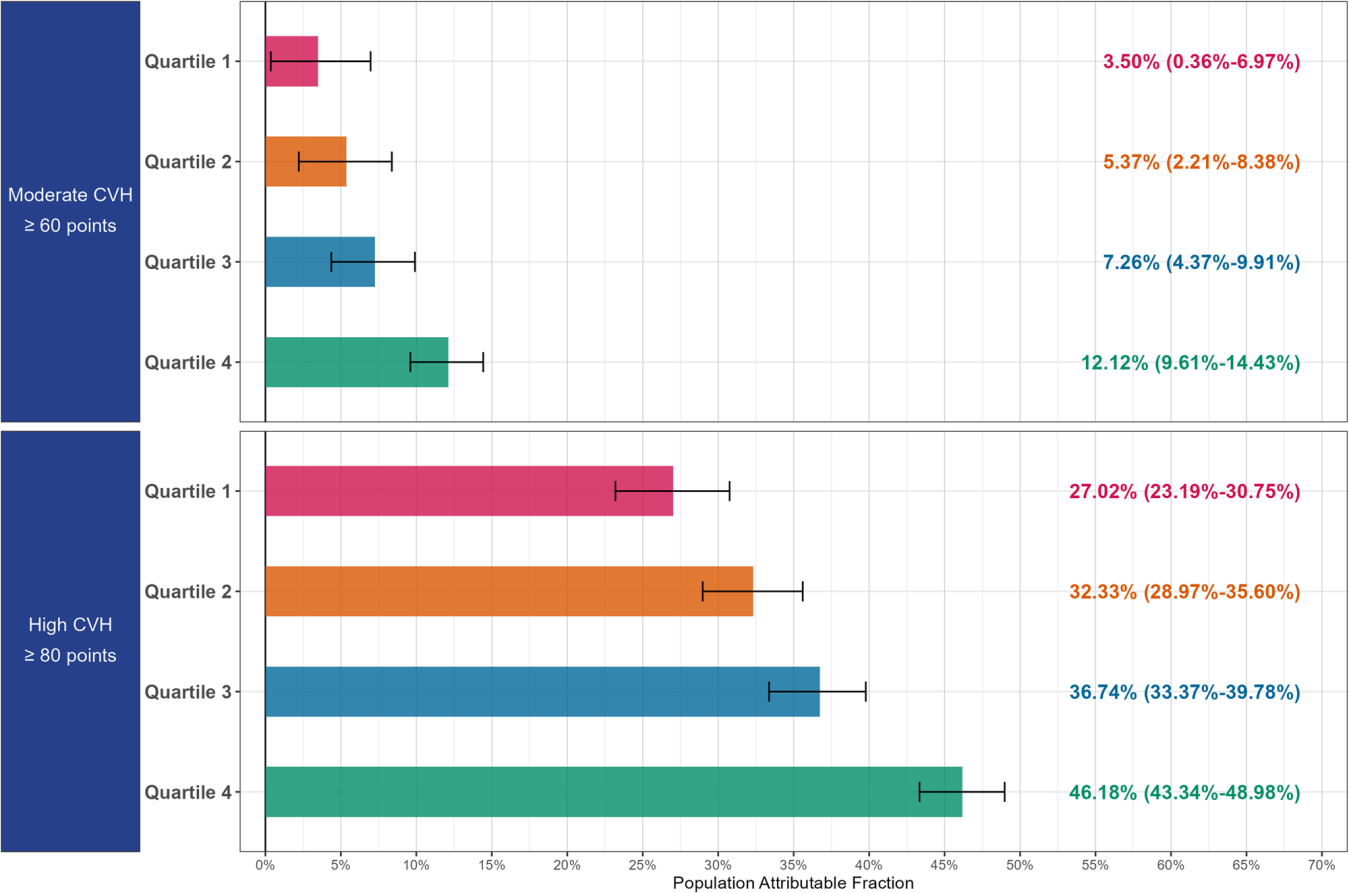
Proportion of preventable major adverse cardiovascular events across metabolic vulnerability index quartiles. The bar charts display the population attributable fraction under two counterfactual scenarios of improved cardiovascular health: increasing Life’s Essential 8 scores to moderate (≥60 points, top panel) and high (≥80 points, bottom panel) levels. Error bars denote the 95% confidence intervals generated by bootstrapping.

We performed counterfactual analyses to estimate the proportion of MACE events that could have been prevented if CVH scores were improved within each MVX quartile (Fig. 2). Under a counterfactual scenario in which all individuals attained at least moderate CVH (LE8 scores ≥ 60), the proportion of potentially preventable MACE cases ranged from 3.50% (95% CI: 0.36%, 6.97%) in Q1 to 12.12% (95% CI: 9.61%, 14.43%) in Q4. The corresponding proportions under a high CVH scenario (LE8 scores ≥ 80) were substantially greater, ranging from 27.02% (95% CI: 23.19%, 30.75%) to 46.18% (95% CI: 43.34%, 48.98%), with the highest potential benefit consistently observed among participants in the highest MVX quartile.

This pattern was replicated across all specific cardiovascular outcomes, with the largest proportion of potentially preventable cases consistently observed among participants in the highest MVX quartile (Supplementary Figs. 10–13). For CVD mortality, preventable fractions ranged from 6.51% to 18.02% under moderate CVH and from 39.63% to 58.01% under high CVH. Similarly, for HF, from 5.00% to 15.12% and from 34.25% to 53.02%; for MI, from 4.49% to 13.37% and from 31.41% to 48.90%; and for stroke, from 2.29% to 9.22% and from 19.90% to 38.48%.

### Subgroup analyses

Subgroup analyses for MACE and the secondary outcomes are shown in Supplementary Figs. 14–18. For MACE, higher MVX was associated with increased risk across most subgroups, with stronger associations in participants aged ≤65 years than in those aged >65 years (Q4 vs Q1, HR 1.26 vs 1.09; *P* for interaction <0.001) and in women than in men (1.44 vs 1.11; *P* for interaction <0.001). Similar sex heterogeneity was observed for the secondary outcomes, with stronger associations in women than in men for MI (1.48 vs 1.27; *P* for interaction <0.001), stroke (1.28 vs 0.96; *P* for interaction <0.001), HF (1.53 vs 0.95; P for interaction < 0.001), and CVD mortality (1.72 vs 1.25; *P* for interaction = 0.004). Age interaction was also evident for MI (1.38 vs 1.16; P for interaction = 0.026) and HF (1.16 vs 1.06; P for interaction = 0.036), and was borderline for CVD mortality (1.43 vs 1.24; P for interaction = 0.056), but was not apparent for stroke (P for interaction = 0.970). By contrast, ethnicity, binge drinking, and CVH category showed limited or outcome-specific heterogeneity. No significant interaction by ethnicity or CVH category was observed for MACE, MI, HF, or CVD mortality, although stroke showed evidence of interaction by ethnicity (P for interaction = 0.027). Interaction by binge drinking was present for MI (P for interaction = 0.041), but not for the other outcomes.

### Incremental predictive performance

We further evaluated the incremental predictive performance of the full model compared with the base model using the C-index, IDI, and NRI. For MACE, the full model showed a small but statistically significant improvement in prediction (C-index: 0.727–0.728; NRI = 4.08%, *P* < 0.001; IDI = 0.02%, *P* < 0.001). Similar modest improvements were observed for MI, HF, and CVD mortality, whereas no meaningful improvement was observed for stroke (Supplementary Table 4).

### Mediation analysis

We further performed mediation analyses to quantify the extent to which MVX mediated the associations between LE8 and cardiovascular outcomes (Supplementary Table 5). For continuous LE8, significant partial mediation by MVX was observed for MACE (4.8%, 95% CI: 3.6%, 6.3%), HF (2.9%, 95% CI: 1.6%, 5.5%), MI (5.6%, 95% CI: 4.1%, 7.6%), and CVD mortality (5.3%, 95% CI: 3.4%, 8.1%), whereas mediation for stroke was not statistically significant. When LE8 was analyzed categorically, mediation proportions were generally larger, ranging from 4.1% to 8.3% across outcomes.

### Individual biomarker contributions to the MVX score

In our biomarker-level analyses, GlycA showed the strongest positive association with MACE, whereas sHDL showed the strongest inverse association (Supplementary Fig. 19). Correlation analyses showed that valine, isoleucine, and leucine, three branched-chain amino acids (BCAAs), formed a strongly correlated BCAA-related domain (r range from 0.84 to 0.91; Supplementary Fig. 20), while GlycA and sHDL loaded predominantly on distinct inflammatory and lipid-related protective factors (loadings 0.95 and 0.98, respectively; Supplementary Table 6). Together, these three factors explained 78.9% of the total variance.

### Sensitivity analyses

The results of the sensitivity analyses are shown in Supplementary Table 7. After excluding participants who experienced events within the first year of follow-up, the association for the highest versus lowest MVX quartile remained significant for MACE (HR = 1.40, 95% CI 1.34, 1.46), stroke (HR = 1.18, 95% CI 1.09, 1.27), HF (HR = 1.31, 95% CI 1.23, 1.40), MI (HR = 1.58, 95% CI 1.48, 1.68), and CVD mortality (HR = 1.63, 95% CI 1.47, 1.81). In the competing-risk framework, the corresponding HRs (95% CI) were 1.19 (1.14, 1.25) for MACE, 1.05 (0.97, 1.12) for stroke, 1.11 (1.04, 1.18) for HF, and 1.31 (1.23, 1.39) for MI. In the imputed dataset, the corresponding HRs (95% CI) were 1.21 (1.15, 1.26) for MACE, 1.06 (0.98, 1.14) for stroke, 1.12 (1.05, 1.20) for HF, 1.32 (1.24, 1.41) for MI, and 1.36 (1.23, 1.51) for CVD mortality. Overall, these sensitivity analyses supported the robustness of the main findings, although the associations for stroke were attenuated in the competing-risk and multiple-imputation analyses.

## Discussion

In this large-scale prospective analysis of 239,135 individuals from the UK Biobank, we observed the following three main findings. First, NMR-derived MVX score is a robust and independent predictor of incident MACE. Conversely, higher CVH, as quantified by the LE8 score, was strongly associated with a reduced risk of MACE. Second, there was a significant interaction between these two metrics, whereby high CVH markedly attenuated the substantial risk conferred by high metabolic vulnerability, individuals with low CVH in the highest MVX quartile faced a 2.84-fold higher MACE risk compared to their counterparts with high CVH in the lowest MVX quartile. Third, our counterfactual analyses estimating that achieving optimal CVH (LE8 ≥ 80) could potentially prevent nearly half of MACE cases among those with the highest underlying metabolic vulnerability. While the primary MACE composite includes outcomes with heterogeneous etiologies, analyses of the individual components showed directionally similar but quantitatively different associations. Elevated MVX was associated with higher risk across all component endpoints, but the associations were strongest for CVD mortality and MI, intermediate for HF, and weakest for stroke. Similarly, higher LE8 was inversely associated with all component endpoints, with the largest relative risk reductions observed for CVD mortality and MI and the smallest for stroke.

While previous studies have examined metabolic markers or lifestyle factors separately, our study is the first large prospective study to comprehensively examine both the independent and combined associations of MVX and LE8 with incident MACE and to quantify the potential population benefit of targeted CVH improvements across different metabolic risk strata.

Metabolic dysregulation is a well-recognized contributor to cardiovascular disease and MACE, partly through chronic inflammation, endothelial dysfunction, and accelerated atherogenesis^12^. While conventional clinical markers such as C-reactive protein (CRP), interleukin-6 (IL-6), and HOMA-IR capture more specific dimensions of inflammation or insulin resistance, the NMR-derived MVX provides a broader metabolomics-based profile of cardiometabolic vulnerability. Its heterogeneous components reflect several intersecting biological domains rather than a single isolated pathway^13–16^.GlycA represents a composite NMR signal of multiple acute-phase glycoproteins and is considered a relatively stable marker of chronic systemic inflammation^15^. Conversely, sHDL may reflect alterations in lipoprotein remodeling and HDL-related lipid transport^17^. Citrate, a key intermediate of the tricarboxylic acid cycle, may indicate perturbation of mitochondrial metabolism^18^. Branched-chain amino acids, including valine, isoleucine, and leucine, have been associated with impaired amino acid catabolism, insulin resistance, and metabolic inflexibility^19,20^. Taken together, a high MVX score may reflect an unfavorable metabolic-inflammatory milieu characterized by persistent inflammation, altered lipid handling, and systemic metabolic stress, which may help explain its association with incident MACE.

Our biomarker-level analyses further improved the biological interpretability of the MVX score. While the composite MVX score provides a holistic assessment of metabolic vulnerability, GlycA showed the strongest positive association with MACE, whereas sHDL showed the strongest inverse association. Correlation and factor analyze further indicated that valine, isoleucine, and leucine clustered strongly together, consistent with a shared BCAA-related metabolic domain, while GlycA and sHDL loaded predominantly on separate inflammatory and lipid-related protective domains. These findings suggest that the predictive signal captured by MVX is driven primarily by three complementary biological dimensions, including systemic inflammation, lipid-related protection, and BCAA-related metabolic dysregulation, rather than by any single isolated pathway.

Another key contribution of this study is the demonstration that the risk associated with a high MVX score is substantially modifiable through improvements in CVH. The CVH factors comprising the LE8 score mitigate the adverse metabolic pathways captured by the MVX biomarkers. For instance, positive behavioral modifications such as adopting a healthy diet and engaging in regular physical activity are established interventions for reducing systemic inflammation^21,22^. Similarly, a suitable lifestyle is known to enhance insulin sensitivity and to promote mitochondrial efficiency, potentially ameliorating the metabolic disorder^23^. Furthermore, the effective management of clinical risk factors integral to the LE8 score, including blood pressure, non-HDL cholesterol, and blood glucose, directly mitigates the downstream vascular damage accelerated by the pro-atherogenic state signified by a high MVX^24^. Consistent with this biological interplay, our mediation analyses further suggested that MVX may partially mediate the associations between LE8 and cardiovascular outcomes, although the mediation proportions were modest overall. These findings indicate that metabolic vulnerability may explain part, but not all, of the cardiovascular risk associated with less favorable CVH.

In addition to the overall joint effects, our subgroup analyses underscore the robustness of these findings across various clinical and demographic strata. Importantly, we observed significant sex heterogeneity in the association between MVX and MACE, with higher risk in female than in male. Given that MVX is a sex-specific standardized score, this finding is biologically plausible and suggests that sex-specific risk stratification and metabolic profiling warrant further targeted investigation. Encouragingly, the ability of favorable CVH to attenuate this metabolic risk appears universally applicable, as the synergistic protective effect of LE8 remained consistent across both sexes and other key subgroups, including differing baseline health statuses.

The variation in effect sizes across the individual MACE endpoints warrants careful consideration. Associations of both MVX and LE8 were strongest for CVD mortality and MI. This pattern may reflect greater sensitivity of these endpoints to the atherosclerotic, inflammatory, lipid-related, and insulin resistance pathways captured by MVX biomarker and influenced by LE8-defined CVH^7^. These mechanisms may be more directly linked to coronary atherosclerosis and fatal cardiovascular events than to more heterogeneous outcomes. By contrast, stroke showed the weakest associations. One plausible explanation is etiologic heterogeneity, because our stroke outcome combined ischemic, hemorrhagic, and unspecified stroke, which differ substantially in their pathophysiology and may not be equally sensitive to metabolic vulnerability or lifestyle-related factors^25^. Differences in statistical power may also have contributed to uncertainty in some estimates. However, statistical power alone is unlikely to fully explain the observed pattern, as CVD mortality showed some of the strongest point estimates despite having fewer events than several nonfatal endpoints. Finally, differential misclassification related to routinely collected ICD-based outcome definitions, particularly for heterogeneous endpoints such as stroke and HF, may also have attenuated some associations^26^.

From a clinical and public health perspective, our findings may have implications for cardiovascular risk stratification. Current risk assessment models, which are based on traditional risk factors such as age, sex, smoking status, and cholesterol levels, remain valuable for population-level prediction, but they may not fully reflect underlying metabolic vulnerability in some individuals. In this context, the MVX score, quantifiable from a single blood sample via high-throughput NMR spectroscopy, may serve as a complementary marker for identifying individuals at elevated cardiovascular risk. In our predictive analyses, incorporation of MVX was associated with modest incremental predictive value for MACE and several other cardiovascular outcomes, although the absolute improvements in discrimination were small. Accordingly, MVX may help identify a subgroup of individuals who appear to be at relatively low risk according to conventional measures but who nonetheless have a less favorable metabolic profile and higher cardiovascular risk. From a translational perspective, individuals with high MVX may benefit most from intensified overall LE8 optimization, particularly in domains relevant to inflammation, lipid metabolism, and metabolic homeostasis. Because GlycA and sHDL emerged as the strongest individual contributors to the MVX signal, these findings indirectly support closer attention to modifiable CVH components that influence inflammatory burden, glycemic control, adiposity, and atherogenic lipid profiles. However, our study was not designed to formally rank the relative importance of each LE8 component, and these findings should therefore be interpreted as informing a personalized domain-based prevention strategy rather than a single-component intervention hierarchy. More importantly, an elevated MVX score should not be viewed as a fixed prognostic determination, but rather as a dynamic, actionable risk signal that warrants prioritized, intensive intervention. Communicating such personalized, quantifiable benefit has the potential to strengthen clinician–patient dialog and motivate sustained engagement in preventive strategies.

The strengths of our study are considerable, including its large sample size, prospective design with long-term follow-up, standardized and high-quality metabolomic profiling, and comprehensive assessment of CVH using the updated LE8 criteria. However, several limitations must be acknowledged. First, the UK Biobank is predominantly composed of White participants and is subject to a healthy volunteer effect, which may limit the generalizability of our findings to more ethnically diverse or higher-risk populations. Although subgroup-specific estimates were generally directionally similar across ethnic groups, the relatively small number of non-White participants limited precision and reduced our ability to detect potential ethnic heterogeneity. Further validation in more diverse and less selected populations is therefore needed. Second, both MVX and LE8 were assessed only at baseline, and thus changes in MVX or CVH during follow-up could not be captured. Reliance on a single baseline measurement may have introduced exposure misclassification over time and may have attenuated the observed associations. Future prospective cohort studies with repeated measurements are needed to validate these findings. Third, despite adjustment for major covariates, residual confounding from unmeasured or incompletely measured lifestyle factors, such as dietary patterns beyond LE8 metrics and alcohol consumption intensity, cannot be ruled out. Finally, our counterfactual analysis, while informative, is based on statistical modeling and assumes that the observed associations are causal; these estimates require validation in future intervention trials.

In conclusion, higher MVX was associated with incident MACE, and more favorable CVH was associated with a substantially lower estimated event burden under modeled scenarios, particularly among metabolically vulnerable individuals.

## Methods

### Study population

This study utilized data from the UK Biobank, a large-scale, prospective cohort that recruited over 500,000 participants aged 40–69 years from across the United Kingdom between 2006 and 2010. Participants provided detailed information via questionnaires, underwent physical assessments, and supplied biological samples at baseline^27^. The UK Biobank has approval from the North West Multi-center Research Ethics Committee (REC reference^16^:/NW/0274). Written informed consent was obtained from all participants prior to their inclusion in the study.

**Fig. 3:**
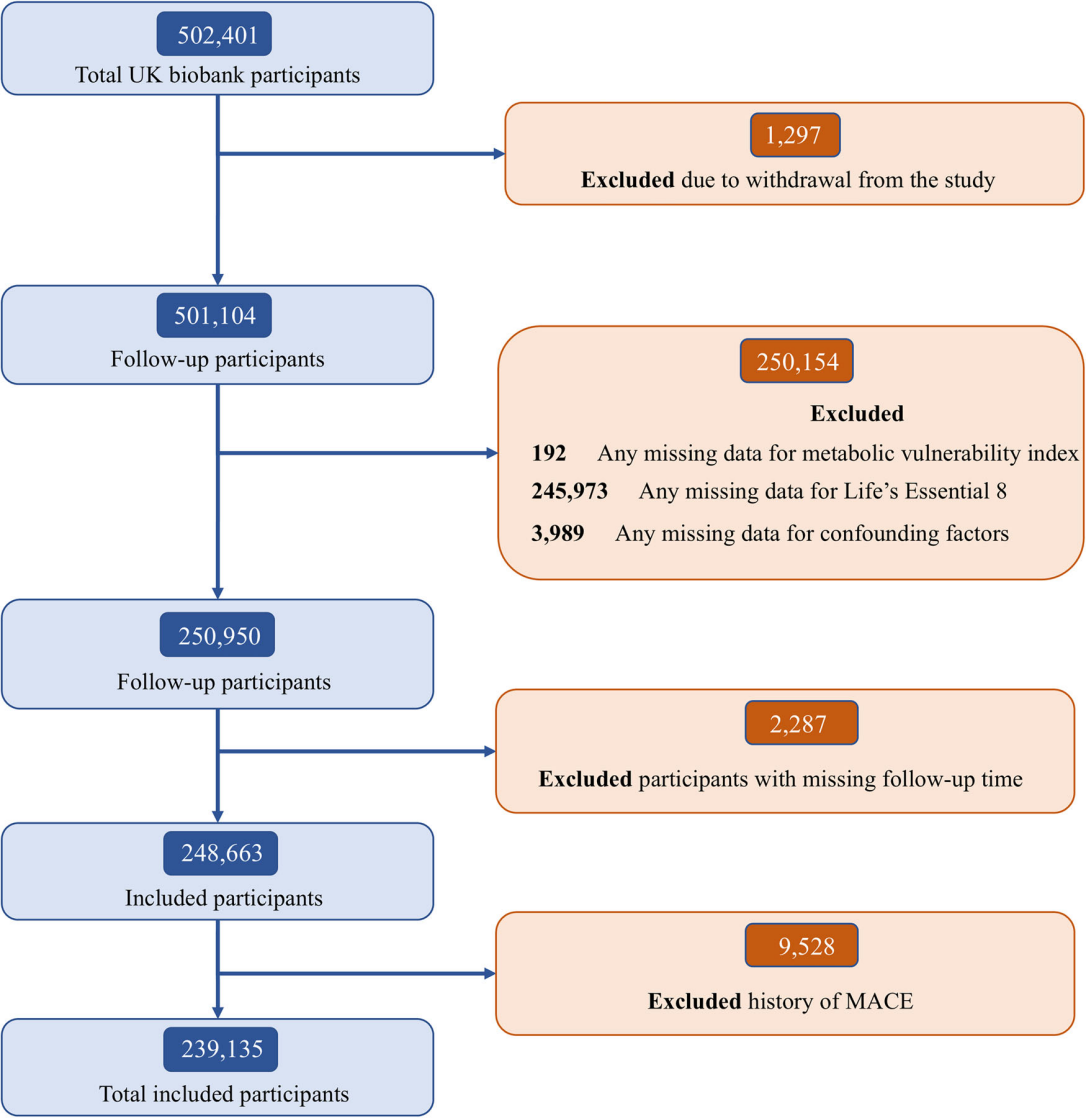
Flowchart of study participant selection. The diagram details the step-by-step inclusion and exclusion process from the initial UK Biobank cohort. Specific reasons for exclusion are outlined, including study withdrawal, missing baseline data (metabolic vulnerability index, Life’s Essential 8, or confounding factors), missing follow-up time, and a history of major adverse cardiovascular event, yielding a final analytical sample of 239,135 participants.

Among the 502,401 recruited individuals, we excluded those who withdrew consent before analysis (*n* = 1297), had missing data on MVX (*n* = 192), LE8 (*n* = 245,973), key covariates (*n* = 3989), or follow-up time (*n* = 2287), or had a history of MACE at baseline (*n* = 9528). The final analytic sample comprised 239,135 participants (Fig. 3).

### Metabolic profiling and MVX measurement

Plasma samples from approximately 280,000 participants underwent baseline metabolic profiling using high-throughput NMR spectroscopy (Nightingale Health platform, Finland), quantifying 251 metabolites. In addition, MVX biomarkers were measured in non-fasting baseline plasma samples, which may introduce some variability in metabolites influenced by recent dietary intake. However, this sampling approach is consistent with the UK Biobank metabolomics resource used in prior study^28^. The MVX score incorporates six specific biomarkers: GlycA, small high-density lipoprotein (sHDL), citrate, valine, isoleucine, and leucine.

As described elsewhere^3^, MVX was calculated using the published sex-specific equations derived in the CATHGEN biorepository. Briefly, the six component metabolites were winsorized at the 1st and 99th percentiles, and sex-specific equations were then used to derive the MVX score. In the original derivation framework, multi-marker scores were normalized using min–max scaling to a range of 1 to 100. In the present study, MVX was standardized within sex (mean = 0, SD = 1) for continuous analyses, such that hazard ratios represent the risk associated with a 1-SD increase in MVX within sex. For categorical analyses, participants were classified into sex-specific quartiles of MVX to account for sex differences in its distribution.

### Ascertainment of LE8

The LE8 scores were calculated according to the American Heart Association’s 2022 updated definition^7^. The LE8 comprises eight components: four behavioral (diet, physical activity, nicotine exposure, sleep health) and four health factors (body mass index, non-HDL cholesterol, blood glucose, and blood pressure). Each component was scored from 0 to 100, and the overall LE8 score was the unweighted average of the eight component scores. Consistent with prior research, the overall LE8 score was categorized into three levels of CVH: low (<60), moderate (60-79), and high (≥80)^8^. More detailed definitions and score information for LE8 metrics can be found in Supplementary Table 8.

### Ascertainment of outcomes

The primary outcome was incident MACE, a composite endpoint of the first occurrence of myocardial infarction (MI), stroke, heart failure (HF) or cardiovascular mortality. Events were identified through linkage to national electronic health records, including Hospital Episode Statistics (for England, Scotland, and Wales) and national death and cancer registries, using International Classification of Diseases (ICD-9 and ICD-10) codes. Detailed ICD codes used for the ascertainment of each MACE component are provided in Supplementary Tables 9–11. Participants were followed from their baseline assessment date until the first MACE event, death, loss to follow-up, or the end of the study period (December 19, 2022, in Scotland; December 8, 2022, in England and Wales), whichever came first.

### Covariates

Demographic and socioeconomic covariates included age, sex (male, female), ethnicity (White, Non-White), and the Townsend deprivation index. The Townsend index, an area-based measure of social deprivation derived from the preceding national census, incorporates unemployment, household overcrowding, non-car ownership, and non-home ownership^29^. Binge drinking was defined as consuming ≥6 standard drinks on a single occasion for women or ≥8 for men. Detailed assessment protocols for alcohol consumption and binge drinking within UK Biobank have been reported previously^30^. Additionally, baseline medication use, including antihypertensive, lipid-lowering, and antidiabetic medications, was ascertained from self-reported medication history.

### Statistical analysis

Baseline characteristics were summarized utilizing appropriate descriptive statistics. Continuous variables were presented as means with standard deviations (SD) or as medians accompanied by interquartile ranges (IQR), while categorical variables were expressed in terms of frequencies and proportions.

To investigate the association between MVX and MACE as well as specific cardiovascular outcomes, Cox proportional hazards regression models were employed. The proportional hazards assumption for all Cox models was assessed using Schoenfeld residuals and diagnostic plots, and no substantial violations were observed (Supplementary Figs. 21–25). MVX was analyzed using two distinct approaches: as a continuous variable (per SD increment) and as a categorical variable based on quartile distributions. Three sequential modeling strategies were implemented: Model 1 adjusted for age and sex; Model 2 was further adjusted for ethnicity, the Townsend deprivation index, binge drinking status, and baseline use of antihypertensive, lipid-lowering, and antidiabetic medications; Model 3 additionally included LE8 category. Hazard ratios (HRs) with corresponding 95% confidence intervals (CIs) were calculated for each modeling approach. Additionally, restricted cubic spline (RCS) models with three knots placed at the 10th, 50th, and 90th percentiles of the exposure distribution were utilized to examine the potential nonlinear exposure-response relationships between MVX and MACE.

To evaluate interaction on the additive scale, we calculated the relative excess risk due to interaction (RERI), attributable proportion due to interaction (AP), and synergy index (SI), each with corresponding 95% confidence intervals. Multiplicative interaction was assessed using likelihood ratio tests comparing models with and without the product interaction term. For interaction analyses, MVX and LE8 were categorized to facilitate clinical interpretation and joint-effect modeling. MVX was categorized into sex-specific quartiles to assess exposure–response gradients without assuming linearity and to maintain balanced group sizes. LE8 was categorized as low (<60), moderate (60–79), and high (≥80) CVH according to standard American Heart Association criteria.

We applied a counterfactual approach to quantify the potential benefit of improving LE8 across MVX strata. Two intervention targets were considered: LE8 ≥ 60 and LE8 ≥ 80, representing at least moderate and high CVH, respectively, in accordance with the American Heart Association LE8 categorization and our previous study^8^. Under each counterfactual scenario, participants with LE8 below the target threshold were reassigned to the threshold value, while those already above the threshold retained their observed values. Predicted outcome incidence under the observed and counterfactual scenarios was then compared to estimate the number and proportion of potentially preventable events. Analyses were performed separately across MVX strata, and 95% confidence intervals were obtained using 1000 bootstrap resamples.

To evaluate the incremental predictive value of the full model beyond the base model, we compared model performance using the C-index, integrated discrimination improvement (IDI), and net reclassification improvement (NRI).

To further clarify the relative contributions of the six biomarkers included in the MVX score, we conducted supplementary biomarker-level analyses using sex-specific standardized values of all six components. Independent associations with incident MACE were assessed by entering all six biomarkers simultaneously into a mutually adjusted Cox proportional hazards model. We then examined pairwise correlations among the biomarkers and performed a factor analysis with Varimax rotation to characterize their latent structure.

We further conducted mediation analyses to quantify the extent to which the MVX score mediated the associations between LE8 and cardiovascular outcomes. *P*-values were adjusted for multiple comparisons using the false discovery rate (FDR) method.

Multiple sensitivity analyses were conducted to assess the stability and validity of the primary findings. A competing risk analytical framework was applied, considering all-cause mortality as a competing event for the primary outcomes of interest. Furthermore, participants with events occurring within the first year of follow-up were excluded to reduce potential reverse causation. Finally, to address potential bias due to missing covariate data, we performed multiple imputation and repeated the primary analyses in the imputed dataset.

All hypothesis testing was conducted using two-sided statistical tests, with statistical significance defined as *P*-values below 0.05.

## Supplementary information

Supplementary information

## Data Availability

The datasets generated and/or analyzed during the current study are not publicly available due to the requirement of UK Biobank’s rigorous approval process for data use.

## References

[1] Conners, K. M. et al. The metabolic vulnerability index: a novel marker for mortality prediction in heart failure. *JACC Heart Fail.* **12**, 290–300 (2024). 37480881 10.1016/j.jchf.2023.06.013 PMC10949384

[2] Totoń-Żurańska, J., Mikolajczyk, T. P., Saju, B. & Guzik, T. J. Vascular remodelling in cardiovascular diseases: hypertension, oxidation, and inflammation. *Clin. Sci.* **138**, 817–850 (2024). 10.1042/CS20220797 38920058

[3] Otvos, J. D. et al. Multimarkers of metabolic malnutrition and inflammation and their association with mortality risk in cardiac catheterisation patients: a prospective, longitudinal, observational, cohort study. *Lancet Healthy Longev.* **4**, e72–e82 (2023). 36738747 10.1016/S2666-7568(23)00001-6

[4] Kunutsor, S. K. et al. Associations of multimarkers of metabolic malnutrition and inflammation with all-cause mortality by multimorbidity status. *Nutrients* **17**, 1747 (2025). 40507016 10.3390/nu17111747 PMC12157198

[5] Xie, R. Metabolomics data improve 10-year cardiovascular risk prediction with the SCORE2 algorithm for the general population without cardiovascular disease or diabetes. *Eur. J. Prevent. Cardiol.* **00**, 1–10 (2025). 10.1093/eurjpc/zwaf254 40269530

[6] Wicks, T. R. et al. Endogenous ketone bodies are associated with metabolic vulnerability and disability in multiple sclerosis. *Nutrients* **17**, 640 (2025). 40004969 10.3390/nu17040640 PMC11858685

[7] Lloyd-Jones, D. M. et al. Life’s essential 8: updating and enhancing the American Heart Association’s construct of cardiovascular health: a presidential advisory from the American Heart Association. *Circulation* **146**, e18–e43 (2022). 35766027 10.1161/CIR.0000000000001078 PMC10503546

[8] Zhang, J. et al. Relation of Life’s Essential 8 to the genetic predisposition for cardiovascular outcomes and all-cause mortality: results from a national prospective cohort. *Eur. J. Prevent. Cardiol.* **30**, 1676–1685 (2023). 10.1093/eurjpc/zwad179 37228091

[9] Yerabolu, K. et al. Cardiovascular health in pregnancy according to Life’s Essential 8 score. *npj Cardiovasc. Health* **3**, 18 (2026). 41922559 10.1038/s44325-026-00117-6 PMC13043774

[10] Guo, J. et al. Central adiposity indices and inflammatory markers mediate the association between life’s crucial 9 and periodontitis in US adults. *Lipids Health Dis.* **24**, 199 (2025). 40462074 10.1186/s12944-025-02619-1 PMC12131473

[11] Walker, J. et al. Cumulative Life’s Essential 8 scores and cardiovascular disease risk. *JAMA Cardiol.* **10**, 649–656 (2025). 40266596 10.1001/jamacardio.2025.0630 PMC12019673

[12] Deprince, A., Haas, J. T. & Staels, B. Dysregulated lipid metabolism links NAFLD to cardiovascular disease. *Mol. Metab.* **42**, 101092 (2020). 33010471 10.1016/j.molmet.2020.101092 PMC7600388

[13] Hu, T. et al. Metabolic regulation of the immune system in health and diseases: mechanisms and interventions. *Signal Transduct. Target Ther.* **9**, 268 (2024). 39379377 10.1038/s41392-024-01954-6 PMC11461632

[14] Svedbom, A. et al. Skin inflammation, systemic inflammation, and cardiovascular disease in psoriasis. *JAMA Dermatol* **161**, 81–86 (2025). 39565616 10.1001/jamadermatol.2024.4433 PMC11579891

[15] Zhang, X. R. et al. Improved prediction and risk stratification of major adverse cardiovascular events using an explainable machine learning approach combining plasma biomarkers and traditional risk factors. *Cardiovasc. Diabetol.* **24**, 153 (2025). 40176039 10.1186/s12933-025-02711-x PMC11966882

[16] Royer, P. et al. Large-scale plasma proteomics in the UK Biobank modestly improves prediction of major cardiovascular events in a population without previous cardiovascular disease. *Eur. J. Prevent. Cardiol.* **31**, 1681–1689 (2024). 10.1093/eurjpc/zwae124 38546334

[17] Rohatgi, A., Westerterp, M., von Eckardstein, A., Remaley, A. & Rye, K. A. HDL in the 21st century: a multifunctional roadmap for future HDL research. *Circulation* **143**, 2293–2309 (2021). 34097448 10.1161/CIRCULATIONAHA.120.044221 PMC8189312

[18] Banach, M. et al. Bempedoic acid in the management of lipid disorders and cardiovascular risk. 2023 position paper of the International Lipid Expert Panel (ILEP). *Prog. Cardiovasc. Dis.* **79**, 2–11 (2023). 36889490 10.1016/j.pcad.2023.03.001

[19] Zhang, X. et al. Leucine accelerates atherosclerosis through dose-dependent MTOR activation in macrophages. *Autophagy* **21**, 1618–1620 (2025). 40047228 10.1080/15548627.2025.2474603 PMC12282986

[20] Wang, Y. et al. Association of circulating branched-chain amino acids with risk of cardiovascular disease: a systematic review and meta-analysis. *Atherosclerosis* **350**, 90–96 (2022). 35576716 10.1016/j.atherosclerosis.2022.04.026

[21] Geng, T. et al. Healthy lifestyle behaviors, mediating biomarkers, and risk of microvascular complications among individuals with type 2 diabetes: a cohort study. *PLoS Med.* **20**, e1004135 (2023). 36626356 10.1371/journal.pmed.1004135 PMC9831321

[22] Valenzuela, P. L. et al. Exercise benefits in cardiovascular diseases: from mechanisms to clinical implementation. *Eur. Heart J.* **44**, 1874–1889 (2023). 37005351 10.1093/eurheartj/ehad170

[23] Niu, M. et al. Emerging healthy lifestyle factors and all-cause mortality among people with metabolic syndrome and metabolic syndrome-like characteristics in NHANES. *J. Transl. Med.* **21**, 239 (2023). 37005663 10.1186/s12967-023-04062-1 PMC10068159

[24] Li, X., Ma, H., Wang, X., Feng, H. & Qi, L. Life’s essential 8, genetic susceptibility, and incident cardiovascular disease: a prospective study. *Arteriosc. Thromb. Vasc. Biol.* **43**, 1324–1333 (2023). 10.1161/ATVBAHA.123.319290 PMC10330462 37199161

[25] O’Donnell, M. J. et al. Risk factors for ischaemic and intracerebral haemorrhagic stroke in 22 countries (the INTERSTROKE study): a case-control study. *Lancet* **376**, 112–123 (2010). 20561675 10.1016/S0140-6736(10)60834-3

[26] Bosco, E., Hsueh, L., McConeghy, K. W., Gravenstein, S. & Saade, E. Major adverse cardiovascular event definitions used in observational analysis of administrative databases: a systematic review. *BMC Med. Res. Methodol.* **21**, 241 (2021). 34742250 10.1186/s12874-021-01440-5 PMC8571870

[27] Zhang, J. et al. The role of life’s essential 8 and multimorbidity in the risk of new-onset atrial fibrillation: observations from a large prospective cohort study. *Int. J. Surg.* **111**, 6885–6893 (2025). 40549419 10.1097/JS9.0000000000002831 PMC12527731

[28] Sun, B. et al. Plasma proteomic associations with genetics and health in the UK Biobank. *Nature* **622**, 329–338 (2023). 37794186 10.1038/s41586-023-06592-6 PMC10567551

[29] Foster, H. M. E. et al. The effect of socioeconomic deprivation on the association between an extended measurement of unhealthy lifestyle factors and health outcomes: a prospective analysis of the UK Biobank cohort. *Lancet Public Health* **3**, e576–e585 (2018). 30467019 10.1016/S2468-2667(18)30200-7

[30] Ding, C. et al. Binge-pattern alcohol consumption and genetic risk as determinants of alcohol-related liver disease. *Nat. Commun.* **14**, 8041 (2023). 38097541 10.1038/s41467-023-43064-x PMC10721893

