# Supplementary material for: Metabolic vulnerability index and Life’s Essential 8 with risk of major adverse cardiovascular events
Source: NPJ Cardiovasc Health. 2026 Jun 12;3:37. doi: 10.1038/s44325-026-00139-0 (PMC13282394; doi:10.1038/s44325-026-00139-0)
Supplement: Supplementary file 1 — Supplementary information [file 44325_2026_139_MOESM1_ESM.pdf]

Supplementary material

Supplementary Table 1. Distribution of population characteristics in individuals included and excluded in the analyses.

	Participants included in analyses (N=239,135)	Participants excluded in analyses* (N=241,475)	Standardized mean differences
Age (years), mean (SD)	55.97 (8.11)	56.71 (8.07)	0.091
Sex (%)			0.130
Female	124897 (52.2)	141585 (58.7)	
Male	114238 (47.8)	99669 (41.3)	
Ethnicity (%)			0.075
Non-white	11158 (4.7)	17451 (6.2)	
White	227977 (95.3)	224024 (93.8)	
Townsend deprivation index, mean±SD	-1.48 (2.98)	-1.17 (3.17)	0.099
Binge drinking			0.027

No	232156 (97.1)	233956 (97.5)	
Yes	6979 (2.9)	5940 (2.5)	
MVX score, mean±SD	27.08 (2.85)	26.74 (2.81)	0.121
GlycA, µmol/L, mean±SD	813.00 (119.44)	812.50 (128.22)	0.004
sHDL, µmol/L, mean±SD	9.87 (1.32)	9.80 (1.43)	0.048
Citrate, µmol/L, mean±SD	66.78 (13.19)	66.34 (13.98)	0.032
Isoleucine, µmol/L, mean±SD	52.06 (17.78)	50.65 (18.17)	0.079
Leucine, µmol/L, mean±SD	105.96 (28.39)	103.05 (29.22)	0.101
Valine, µmol/L, mean±SD	212.67 (43.10)	208.51 (44.83)	0.095
AHA LE8 Scores (out of 100 possible points), mean±SD			
Mean Total CVH Score	69.95 (11.53)	69.80 (11.81)	0.013
Dietary recommendations for cardiovascular health	62.13 (17.43)	61.16 (17.86)	0.055
Physical activity	77.94 (36.39)	78.05 (36.40)	0.003

Tobacco/nicotine exposure	76.61 (31.56)	75.46 (32.95)	0.036
Sleep health	89.72 (18.22)	88.57 (19.40)	0.061
Body mass index	70.23 (28.00)	68.23 (29.08)	0.07
Blood lipids (non-HDL cholesterol)	47.72 (28.97)	47.02 (29.05)	0.024
Blood glucose	91.88 (18.54)	90.99 (19.33)	0.047
Blood pressure	43.38 (32.36)	41.60 (32.46)	0.055
**LE8 score stratification**			0.032
High CVH (80~100)	48639 (20.3)	717 (21.0)	
Moderate CVH (60~79)	146283 (61.2)	2036 (59.6)	
Low CVH (0~59)	44213 (18.5)	661 (19.4)	

*Percentages among excluded participants were calculated using variable-specific non-missing denominators; therefore, counts may not sum to the total number of excluded participants. Standardized mean differences were calculated using available data for each variable.

Supplementary Table 2. Associations between Life's Essential 8 (LE8) and incident MACE.

	Cases (%)	IR (95% CI) per 1000 person-years	HR (95% CI)	*P* value
**MACE**				
Per 1-point increase	17146/239135 (7.17%)	5.46 (5.38, 5.54)	0.97 (0.97, 0.97)	<.0001
Low CVH	5503/44213 (12.45%)	9.81 (9.55, 10.07)	Ref.	
Moderate CVH	10117/146283 (6.92%)	5.26 (5.16, 5.36)	0.64 (0.62, 0.67)	<.0001
High CVH	1526/48639 (3.14%)	2.33 (2.21, 2.44)	0.44 (0.41, 0.47)	<.0001
**Stroke**				
Per 1-point increase	6017/239135 (2.52%)	1.89 (1.84, 1.94)	0.98 (0.98, 0.98)	<.0001
Low CVH	1736/44213 (3.93%)	3.02 (2.88, 3.16)	Ref.	
Moderate CVH	3625/146283 (2.48%)	1.86 (1.80, 1.92)	0.70 (0.66, 0.74)	<.0001
High CVH	656/48639 (1.35%)	0.99 (0.92, 1.07)	0.55 (0.50, 0.61)	<.0001
**HF**				
Per 1-point increase	7301/239135 (3.05%)	2.29 (2.24, 2.35)	0.97 (0.97, 0.97)	<.0001

Low CVH	2588/44213 (5.85%)	4.51 (4.34, 4.69)	Ref.	
Moderate CVH	4147/146283 (2.83%)	2.13 (2.06, 2.19)	0.60 (0.57, 0.63)	<.0001
High CVH	566/48639 (1.16%)	0.86 (0.79, 0.93)	0.42 (0.38, 0.46)	<.0001
**MI**				
Per 1-point increase	8390/239135 (3.51%)	2.65 (2.59, 2.70)	0.97 (0.97, 0.97)	<.0001
Low CVH	2828/44213 (6.40%)	4.97 (4.79, 5.16)	Ref.	
Moderate CVH	4936/146283 (3.37%)	2.54 (2.47, 2.61)	0.63 (0.60, 0.66)	<.0001
High CVH	626/48639 (1.29%)	0.95 (0.88, 1.02)	0.37 (0.34, 0.40)	<.0001
**CVD mortality**				
Per 1-point increase	3225/239135 (1.35%)	1.00 (0.97, 1.04)	0.96 (0.96, 0.97)	<.0001
Low CVH	1233/44213 (2.79%)	2.12 (2.00, 2.24)	Ref.	
Moderate CVH	1773/146283 (1.21%)	0.90 (0.86, 0.94)	0.56 (0.52, 0.60)	<.0001
High CVH	219/48639 (0.45%)	0.33 (0.29, 0.37)	0.36 (0.31, 0.42)	<.0001

Supplementary Table 3. Distribution of participants and incidence of cardiovascular outcomes across joint categories of cardiovascular health status and MVX quartile.

Cardiovascular health status	MVX quartile	Total Participants, n	MACE, n (%)	Stroke, n (%)	HF, n (%)	MI, n (%)	CVD mortality, n (%)
High CVH	Q1	19,864	588 (2.96%)	274 (1.38%)	222 (1.12%)	231 (1.16%)	85 (0.43%)
	Q2	13,393	412 (3.08%)	171 (1.28%)	149 (1.11%)	176 (1.31%)	61 (0.46%)
	Q3	9,448	317 (3.36%)	124 (1.31%)	109 (1.15%)	144 (1.52%)	39 (0.41%)
	Q4	5,934	209 (3.52%)	87 (1.47%)	86 (1.45%)	75 (1.26%)	34 (0.57%)
Moderate CVH	Q1	37,372	2,310 (6.18%)	849 (2.27%)	970 (2.60%)	1,081 (2.89%)	365 (0.98%)
	Q2	37,452	2,510 (6.70%)	949 (2.53%)	1,036 (2.77%)	1,191 (3.18%)	448 (1.20%)
	Q3	36,751	2,687 (7.31%)	933 (2.54%)	1,084 (2.95%)	1,349 (3.67%)	482 (1.31%)
	Q4	34,708	2,610 (7.52%)	894 (2.58%)	1,057 (3.05%)	1,315 (3.79%)	478 (1.38%)

Low CVH	Q1	5,992	631 (10.53%)	231 (3.86%)	331 (5.52%)	295 (4.92%)	140 (2.34%)
	Q2	8,834	1,016 (11.50%)	332 (3.76%)	468 (5.30%)	542 (6.14%)	215 (2.43%)
	Q3	11,709	1,468 (12.54%)	456 (3.89%)	685 (5.85%)	760 (6.49%)	325 (2.78%)
	Q4	17,678	2,388 (13.51%)	717 (4.06%)	1,104 (6.25%)	1,231 (6.96%)	553 (3.13%)

Supplementary Table 4. Incremental predictive value of adding the MVX score to the baseline clinical model.

**Outcome**	**Base Model C-index**	**Full Model C-index**	**Δ C-index**	**IDI (%)**	P_{IDI}	**NRI (%)**	P_{NRI}
MACE	0.7273	0.7284	0.0011	0.02	<0.001	4.08	<0.001
Stroke	0.7108	0.7111	0.0003	0.00	0.56	-0.63	0.64
Myocardial Infarction	0.7374	0.7388	0.0014	0.02	0.02	6.13	<0.001
Heart Failure	0.7813	0.7817	0.0004	0.01	0.20	3.94	0.02
CVD mortality	0.7824	0.7839	0.0014	0.01	0.14	7.50	0.02

Supplementary Table 5. Mediation proportion of Life's Essential 8 with outcomes attributed to MVX.

Characteristics	MVX		
	**Mediation proportion (95% CI)**	**P-value**	**FDR-adjusted P-value[a]**
**MACE**			
Per 1-point increase	4.8% (3.6%, 6.3%)	<0.0001	0.0001
Low CVH			
Moderate CVH	6.7% (5.4%, 8.3%)	<0.0001	0.0001
High CVH	8.3% (6.4%, 10.7%)	<0.0001	0.0001
**Stroke**			
Per 1-point increase	2.0% (0.0%, 8.8%)	0.1020	0.1020
Low CVH			
Moderate CVH	4.1% (1.9%, 8.4%)	0.0032	0.0037
High CVH	5.2% (1.9%, 13.6%)	0.0245	0.0263
**HF**			
Per 1-point increase	2.9% (1.6%, 5.5%)	0.0009	0.0011
Low CVH			
Moderate CVH	4.6% (3.1%, 6.8%)	<0.0001	0.0001
High CVH	7.0% (4.4%, 10.8%)	<0.0001	0.0001
**MI**			
Per 1-point increase	5.6% (4.1%, 7.6%)	<0.0001	0.0001
Low CVH			
Moderate CVH	8.0% (6.3%, 10.3%)	<0.0001	0.0001
High CVH	8.0% (5.8%, 10.9%)	<0.0001	0.0001
**CVD mortality**			

Per 1-point increase	5.3% (3.4%, 8.1%)	<0.0001	0.0001
Low CVH			
Moderate CVH	7.3% (5.2%, 10.1%)	<0.0001	0.0001
High CVH	8.3% (5.2%, 12.9%)	<0.0001	0.0001

[a] P-values were adjusted for multiple comparisons using the False Discovery Rate (FDR) method.

Supplementary Table 6. Varimax-rotated factor loadings of the six biomarkers included in the MVX score

**Biomarker**	**Factor 1 BCAA-related metabolic domain**	**Factor 2 Lipid-related protective domain**	**Factor 3 Inflammatory domain**
GlycA	0.187	0.225	**0.954**
sHDL	0.047	**0.981**	0.174
Citrate	0.161	0.15	0.102
Valine	**0.879**	0.137	0.223
Isoleucine	**0.925**	0.028	0.122
Leucine	**0.974**	0.121	0.089

Note: Values represent factor loadings from exploratory factor analysis with Varimax rotation. Bold indicates the dominant loading for each biomarker.

Supplementary Table 7. Sensitivity analyses of association between MVX quartile and cardiovascular outcomes.

Characteristics	MACE HR (95% CI)	*P* value	Stroke HR (95% CI)[a]	*P* value	HF HR (95% CI)[a]	*P* value	MI HR (95% CI)[a]	*P* value	CVD mortality HR (95% CI)[a]	*P* value
**Sensitivity analysis 1[a]**										
**MVX quartile**										
per 1-SD increase	1.14 (1.12, 1.16)	<.0001	1.08 (1.05, 1.11)	<.0001	1.13 (1.10, 1.15)	<.0001	1.17 (1.15, 1.20)	<.0001	1.19 (1.16, 1.23)	<.0001
Q1	-		-		-		-		-	
Q2	1.11 (1.06, 1.16)	<.0001	1.05 (0.98, 1.13)	0.1871	1.04 (0.97, 1.12)	0.2228	1.20 (1.12, 1.28)	<.0001	1.20 (1.08, 1.34)	0.0011
Q3	1.22 (1.17, 1.28)	<.0001	1.07 (0.99, 1.15)	0.0929	1.13 (1.05, 1.21)	0.0005	1.38 (1.29, 1.47)	<.0001	1.33 (1.20, 1.48)	<.0001

Q4	1.40 (1.34, 1.46)	<.0001	1.18 (1.09, 1.27)	<.0001	1.31 (1.23, 1.40)	<.0001	1.58 (1.48, 1.68)	<.0001	1.63 (1.47, 1.81)	<.0001
**Sensitivity analysis 2[b]**										
**MVX quartile**										
per 1-SD increase	1.08 (1.06, 1.09)	<.0001	1.03 (1.00, 1.06)	0.0232	1.06 (1.04, 1.09)	<.0001	1.10 (1.08, 1.12)	<.0001	-	
Q1	-		-		-		-		-	
Q2	1.05 (1.00, 1.09)	0.055	1.01 (0.94, 1.09)	0.7388	0.98 (0.92, 1.05)	0.6415	1.11 (1.04, 1.19)	0.0015	-	
Q3	1.11 (1.06, 1.16)	<.0001	1.00 (0.93, 1.08)	0.9614	1.03 (0.96, 1.10)	0.4706	1.22 (1.14, 1.30)	<.0001	-	
Q4	1.19 (1.14, 1.25)	<.0001	1.05 (0.97, 1.12)	0.234	1.11 (1.04, 1.18)	0.0028	1.31 (1.23, 1.39)	<.0001	-	

**Sensitivity analysis 3[c]**										
**MVX quartile**										
per 1-SD increase	1.08 (1.07, 1.10)	<.0001	1.04 (1.01, 1.06)	0.0076	1.06 (1.04, 1.09)	<.0001	1.10 (1.08, 1.13)	<.0001	1.12 (1.08, 1.16)	<.0001
Q1	-		-		-		-		-	
Q2	1.05 (1.00, 1.10)	0.0392	1.01 (0.94, 1.09)	0.7236	0.99 (0.92, 1.06)	0.7442	1.12 (1.05, 1.20)	0.001	1.12 (1.00, 1.25)	0.0455
Q3	1.12 (1.07, 1.17)	<.0001	1.00 (0.93, 1.08)	0.8989	1.03 (0.96, 1.10)	0.3873	1.22 (1.15, 1.30)	<.0001	1.19 (1.07, 1.32)	0.0014
Q4	1.21 (1.15, 1.26)	<.0001	1.06 (0.98, 1.14)	0.1268	1.12 (1.05, 1.20)	0.0008	1.32 (1.24, 1.41)	<.0001	1.36 (1.23, 1.51)	<.0001

[a] In sensitivity analysis 1, participants with events occurring within the first year of follow-up were excluded to reduce potential reverse causation.

[b] In sensitivity analysis 2, competing risks models were used, with all-cause mortality as the competing event.

[c] In sensitivity analysis 3, imputed dataset was used.

Supplementary Table 8. Quantitative Assessment of Life's Essential 8 (LE8).

LE8 metric	Method of measurement	Quantification of LE8 metric	Score and classification
Diet	Measurement: A more recent definition of ideal intake of dietary components for cardiovascular health	Scoring:<br>Points diets score (points)<br>100 8–10<br>80 6–7<br>50 4–5<br>25 2–3<br>0 0–1	The LE8 score is scaled from 0 to 100 points, calculated as the unweighted average of all 8 component metric scores. In the present study, overall LE8 scores of 80 to 100 are considered High CVH; 60 to 79, Moderate CVH; and 0 to 59 points, low CVH.
Physical activity (PA)	Self-reported minutes of moderate or vigorous PA per week	Scoring:<br>Points Minutes<br>100 ≥150<br>90 120–149<br>80 90–119	

		60 60–89<br><br>40 30–59<br><br>20 1–29<br><br>0 0	
Tobacco/nicotine exposure	Self-reported use of cigarettes; or secondhand smoke exposure	Scoring:<br><br>Points Status<br><br>100 Never smoker<br><br>75 Former smoker, quit ≥5 y<br><br>50 Former smoker, quit 1–<5 y<br><br>25 Former smoker, quit <1 y<br><br>0 Current smoker<br><br>Subtract 20 points (unless score is 0) for<br><br>living with active indoor smoker in home	

Sleep health	Self-reported average hours of sleep per night	Scoring:<br>Points Level<br>100 7–<9<br>90 9–<10<br>70 6–<7<br>40 5–<6 or ≥10<br>20 4–<5<br>0 <4	
Body mass index	Measurement: Body weight (kilograms) divided by height squared (meters squared)	Scoring:<br>Points Level<br>100 <25<br>70 25.0–29.9<br>30 30.0–34.9<br>15 35.0–39.9<br>0 ≥40.0	

Blood lipids (non-HDL cholesterol)	Measurement: Plasma total and HDL cholesterol with calculation of non–HDL cholesterol	Metric: Non–HDL cholesterol (mg/dL)<br>Scoring:<br>Points Level<br>100 <130<br>60 130–159<br>40 160–189<br>20 190–219<br>0 ≥220<br>If drug-treated level, subtract 20 points	
Blood glucose	Measurement: HbA1c and history of Diabetes	Metric: HbA1c (%)<br>Scoring:<br>Points Level	

		100 No history of diabetes HbA1c <5.7<br>60 No diabetes and HbA1c 5.7–6.4 (prediabetes)<br>40 Diabetes with HbA1c <7.0<br>30 Diabetes with HbA1c 7.0–7.9<br>20 Diabetes with HbA1c 8.0–8.9<br>10 Diabetes with Hb A1c 9.0–9.9<br>0 Diabetes with HbA1c ≥10.0	
Blood pressure (BP)	Measurement: Appropriately measured systolic and diastolic blood pressures	Metric: Systolic and diastolic BPs (mm Hg)<br>Scoring:<br>Points Level<br>100 <120/<80 (optimal)<br>75 120–129/<80 (elevated)	

		50 130–139 or 80–89 (stage 1 hypertension)<br>25 140–159 or 90–99<br>0 ≥160 or ≥100<br>Subtract 20 points if treated level	

Supplementary Table 9. Stroke code lists.

Code Type	Code	Text
ICD 9 Code	430.X	Subarachnoid haemorrhage
ICD 9 Code	431.X	Intracerebral haemorrhage
ICD 9 Code	434.X	Occlusion of cerebral arteries
ICD 9 Code	434.0	Cerebral thrombosis
ICD 9 Code	434.1	Cerebral embolism
ICD 9 Code	434.9	Cerebral artery occlusion, unspecified
ICD 9 Code	436.X	Acute, but ill-defined, cerebrovascular disease
ICD 10 Code	I60	Subarachnoid haemorrhage
ICD 10 Code	I60.0	Subarachnoid haemorrhage from carotid siphon and bifurcation

ICD 10 Code	I60.1	Subarachnoid haemorrhage from middle cerebral artery
ICD 10 Code	I60.2	Subarachnoid haemorrhage from anterior communicating artery
ICD 10 Code	I60.3	Subarachnoid haemorrhage from posterior communicating artery
ICD 10 Code	I60.4	Subarachnoid haemorrhage from basilar artery
ICD 10 Code	I60.5	Subarachnoid haemorrhage from vertebral artery
ICD 10 Code	I60.6	Subarachnoid haemorrhage from other intracranial arteries
ICD 10 Code	I60.7	Subarachnoid haemorrhage from intracranial artery, unspecified
ICD 10 Code	I60.8	Other subarachnoid haemorrhage
ICD 10 Code	I60.9	Subarachnoid haemorrhage, unspecified
ICD 10 Code	I61	Intracerebral haemorrhage
ICD 10 Code	I61.0	Intracerebral haemorrhage in hemisphere, subcortical

ICD 10 Code	I61.1	Intracerebral haemorrhage in hemisphere, cortical
ICD 10 Code	I61.2	Intracerebral haemorrhage in hemisphere, unspecified
ICD 10 Code	I61.3	Intracerebral haemorrhage in brainstem
ICD 10 Code	I61.4	Intracerebral haemorrhage in cerebellum
ICD 10 Code	I61.5	Intracerebral haemorrhage, intraventricular
ICD 10 Code	I61.6	Intracerebral haemorrhage, multiple localized
ICD 10 Code	I61.8	Other intracerebral haemorrhage
ICD 10 Code	I61.9	Intracerebral haemorrhage, unspecified
ICD 10 Code	I63	Cerebral infarction
ICD 10 Code	I63.0	Cerebral infarction due to thrombosis of precerebral arteries
ICD 10 Code	I63.1	Cerebral infarction due to embolism of precerebral arteries

ICD 10 Code	I63.2	Cerebral infarction due to unspecified occlusion or stenosis of precerebral arteries
ICD 10 Code	I63.3	Cerebral infarction due to thrombosis of cerebral arteries
ICD 10 Code	I63.4	Cerebral infarction due to embolism of cerebral arteries
ICD 10 Code	I63.5	Cerebral infarction due to unspecified occlusion or stenosis of cerebral arteries
ICD 10 Code	I63.6	Cerebral infarction due to cerebral venous thrombosis, nonpyogenic
ICD 10 Code	I63.8	Other cerebral infarction
ICD 10 Code	I63.9	Cerebral infarction, unspecified
ICD 10 Code	I64.X	Stroke, not specified as haemorrhage or infarction

Supplementary Table 10. Myocardial infarction code lists.

Code Type	Code	Text
ICD 9 Code	410	Acute myocardial infarction
ICD 9 Code	410.0	Acute myocardial infarction of anterolateral wall
ICD 9 Code	410.1	Acute myocardial infarction of other anterior wall
ICD 9 Code	410.2	Acute myocardial infarction of inferolateral wall
ICD 9 Code	410.3	Acute myocardial infarction of inferoposterior wall
ICD 9 Code	410.4	Acute myocardial infarction of other inferior wall
ICD 9 Code	410.5	Acute myocardial infarction of other lateral wall
ICD 9 Code	410.6	True posterior wall infarction
ICD 9 Code	410.7	Subendocardial infarction

ICD 9 Code	410.8	Acute myocardial infarction of other specified sites
ICD 9 Code	410.9	Acute myocardial infarction of unspecified site
ICD 9 Code	411.0	Postmyocardial infarction syndrome
ICD 9 Code	412.X	Old myocardial infarction
ICD 9 Code	429.79	Ill-defined descriptions and complications of heart disease – Other
ICD 10 Code	I21	Acute myocardial infarction
ICD 10 Code	I21.0	Acute transmural myocardial infarction of anterior wall
ICD 10 Code	I21.1	Acute transmural myocardial infarction of inferior wall
ICD 10 Code	I21.2	Acute transmural myocardial infarction of other sites
ICD 10 Code	I21.3	Acute transmural myocardial infarction of unspecified site
ICD 10 Code	I21.4	Acute subendocardial myocardial infarction

ICD 10 Code	I21.9	Acute myocardial infarction, unspecified
ICD 10 Code	I22	Subsequent myocardial infarction
ICD 10 Code	I22.0	Subsequent myocardial infarction of anterior wall
ICD 10 Code	I22.1	Subsequent myocardial infarction of inferior wall
ICD 10 Code	I22.8	Subsequent myocardial infarction of other sites
ICD 10 Code	I22.9	Subsequent myocardial infarction of unspecified site
ICD 10 Code	I23	Certain current complications following acute myocardial infarction
ICD 10 Code	I23.0	Haemopericardium as current complication following acute myocardial infarction
ICD 10 Code	I23.1	Atrial septal defect as current complication following acute myocardial infarction
ICD 10 Code	I23.2	Ventricular septal defect as current complication following acute myocardial infarction

ICD 10 Code	I23.3	Rupture of cardiac wall without haemopericardium as current complication following acute myocardial infarction
ICD 10 Code	I23.4	Rupture of chordaetendineae as current complication following acute myocardial infarction
ICD 10 Code	I23.5	Rupture of papillary muscle as current complication following acute myocardial infarction
ICD 10 Code	I23.6	Thrombosis of atrium, auricular appendage, and ventricle as current complications following acute myocardial infarction
ICD 10 Code	I23.8	Other current complications following acute myocardial infarction
ICD 10 Code	I24.1	Dressler syndrome
ICD 10 Code	I25.2	Old myocardial infarction

Supplementary Table 11. Heart failure code lists.

Code Type	Code	Text
ICD 9 Code	428.X	Heart failure
ICD 9 Code	428.0	Congestive heart failure, unspecified
ICD 9 Code	428.1	Left heart failure
ICD 9 Code	428.2	Systolic heart failure
ICD 9 Code	428.3	Diastolic heart failure
ICD 9 Code	428.4	Combined systolic and diastolic heart failure
ICD 9 Code	428.9	Heart failure, unspecified
ICD 10 Code	I50	Heart failure
ICD 10 Code	I50.0	Congestive heart failure

ICD 10 Code	I50.1	Left ventricular failure
ICD 10 Code	I50.9	Heart failure, unspecified

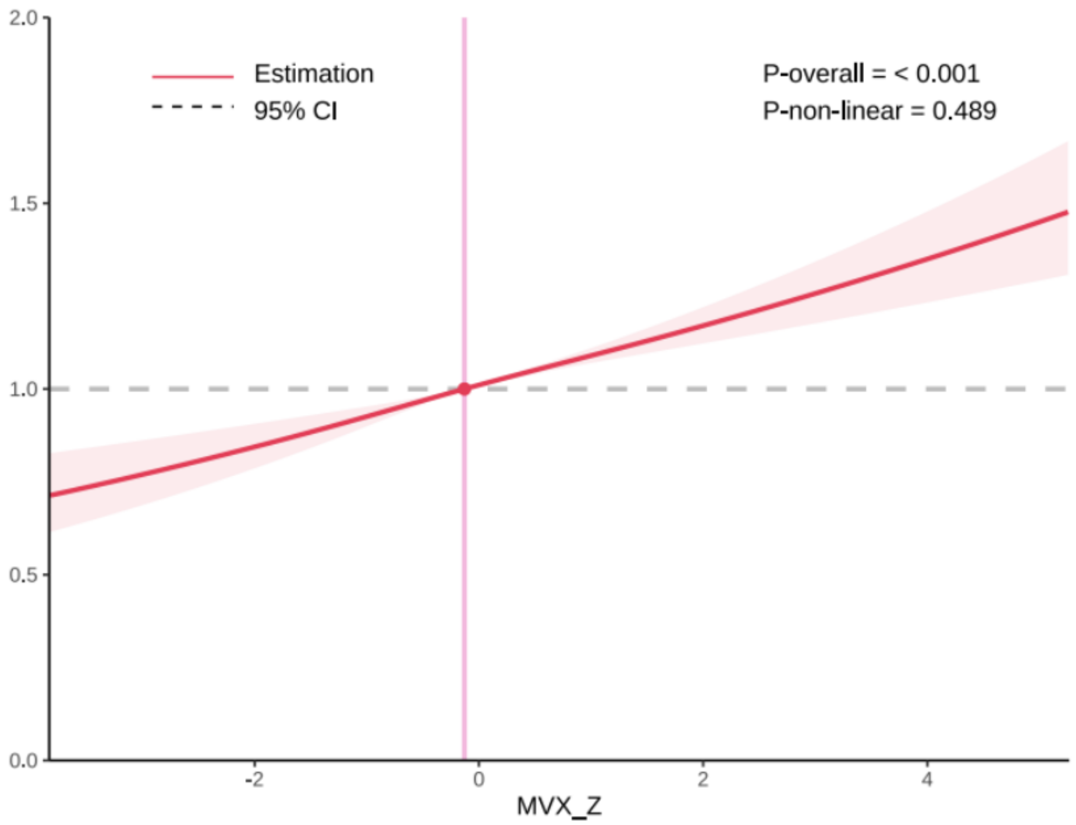

Supplementary Figure 1. Restricted cubic spline curve for the association between MVX and incident MACE.

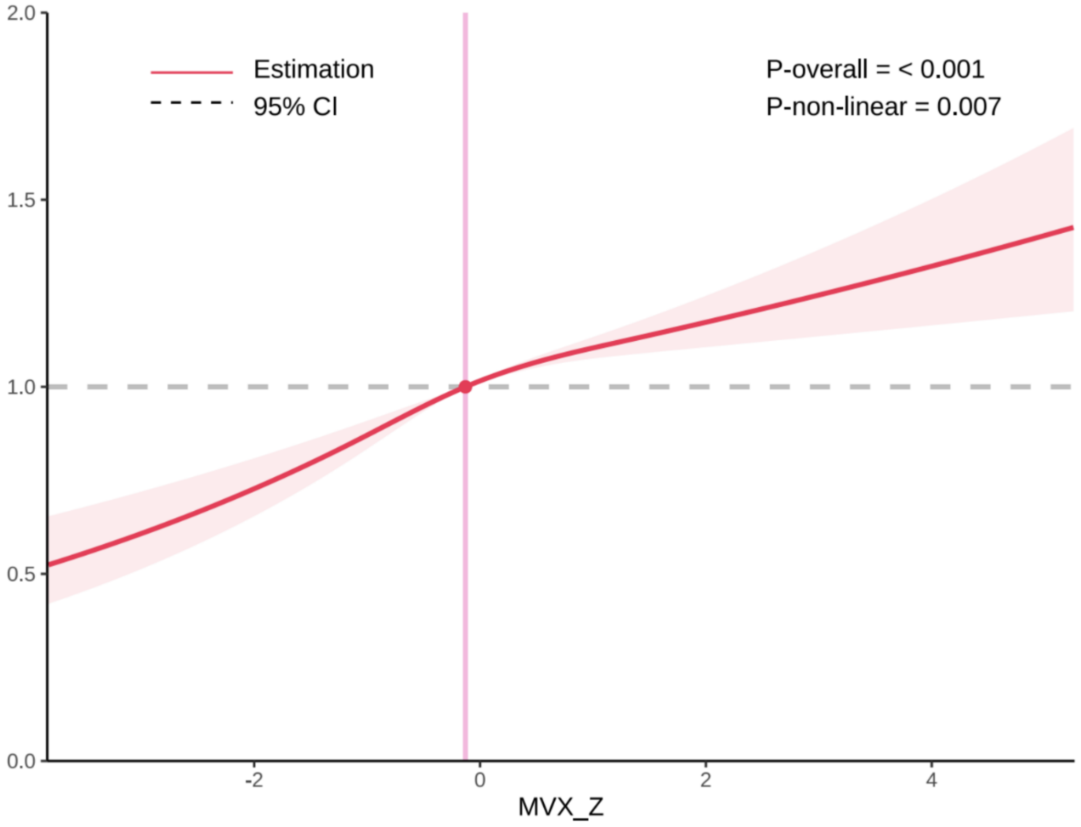

Supplementary Figure 2. Restricted cubic spline curves of the associations between MVX and myocardial infarction.

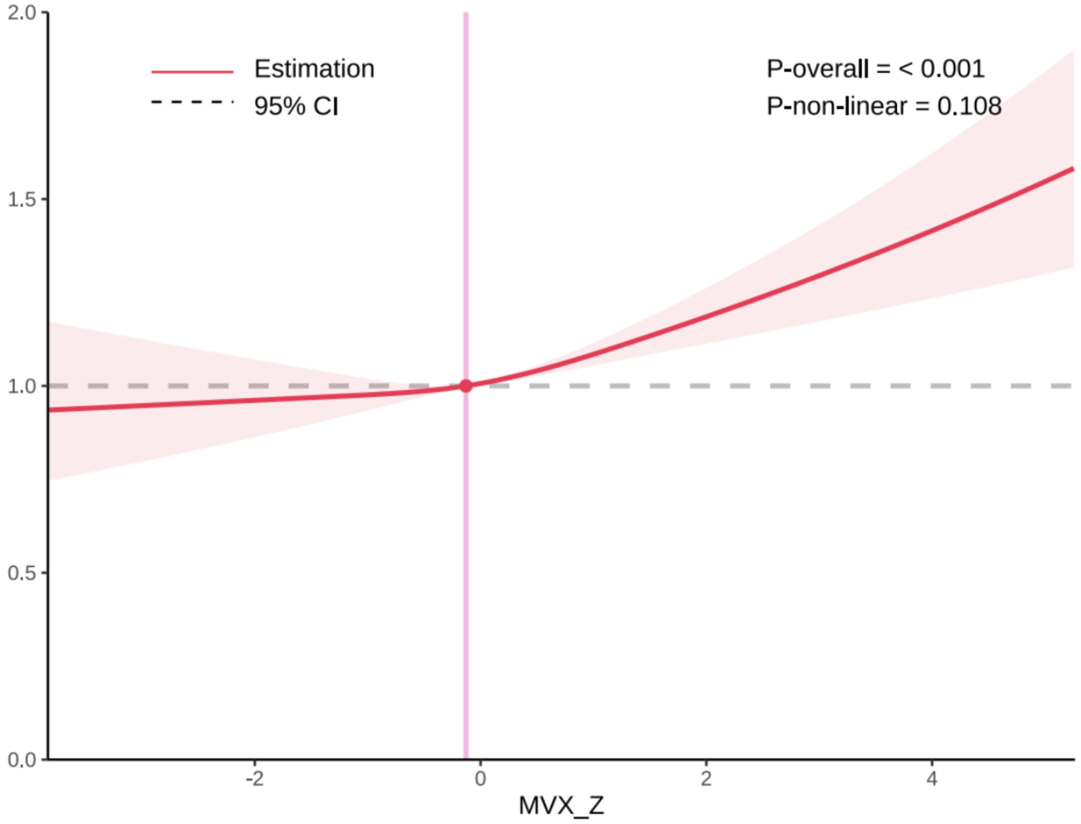

Supplementary Figure 3. Restricted cubic spline curves of the associations between MVX and heart failure.

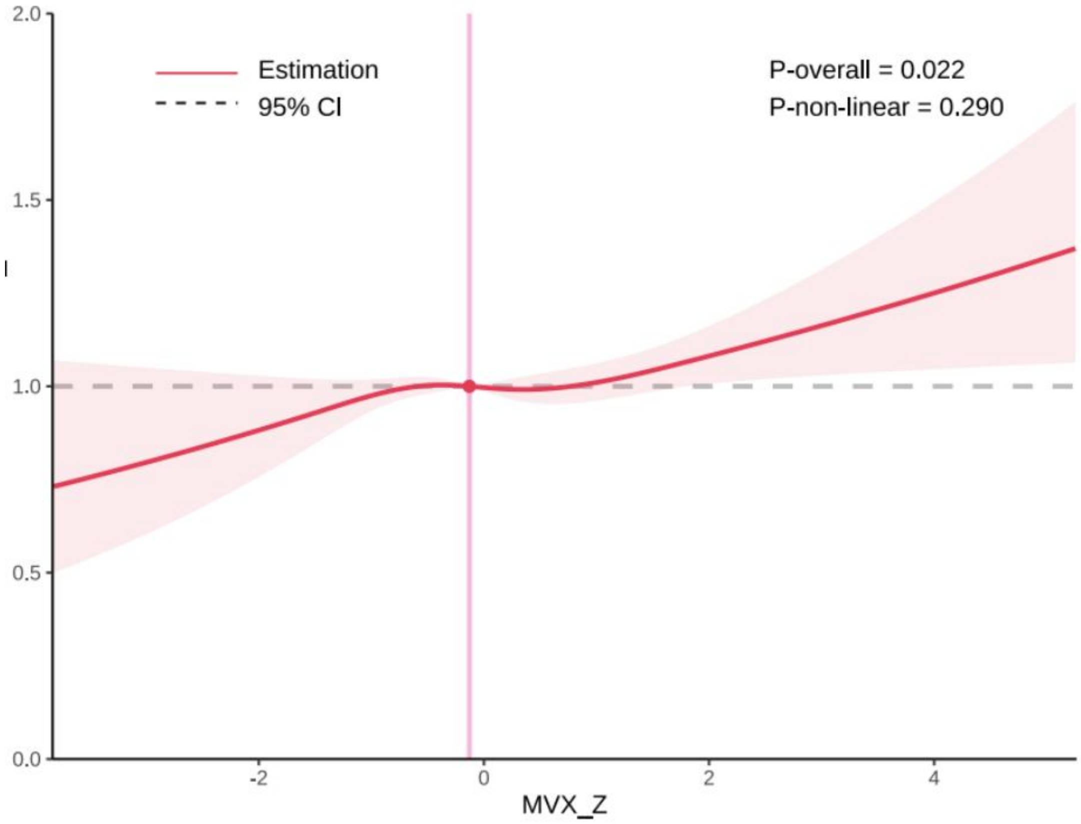

Supplementary Figure 4. Restricted cubic spline curves of the associations between MVX and stroke.

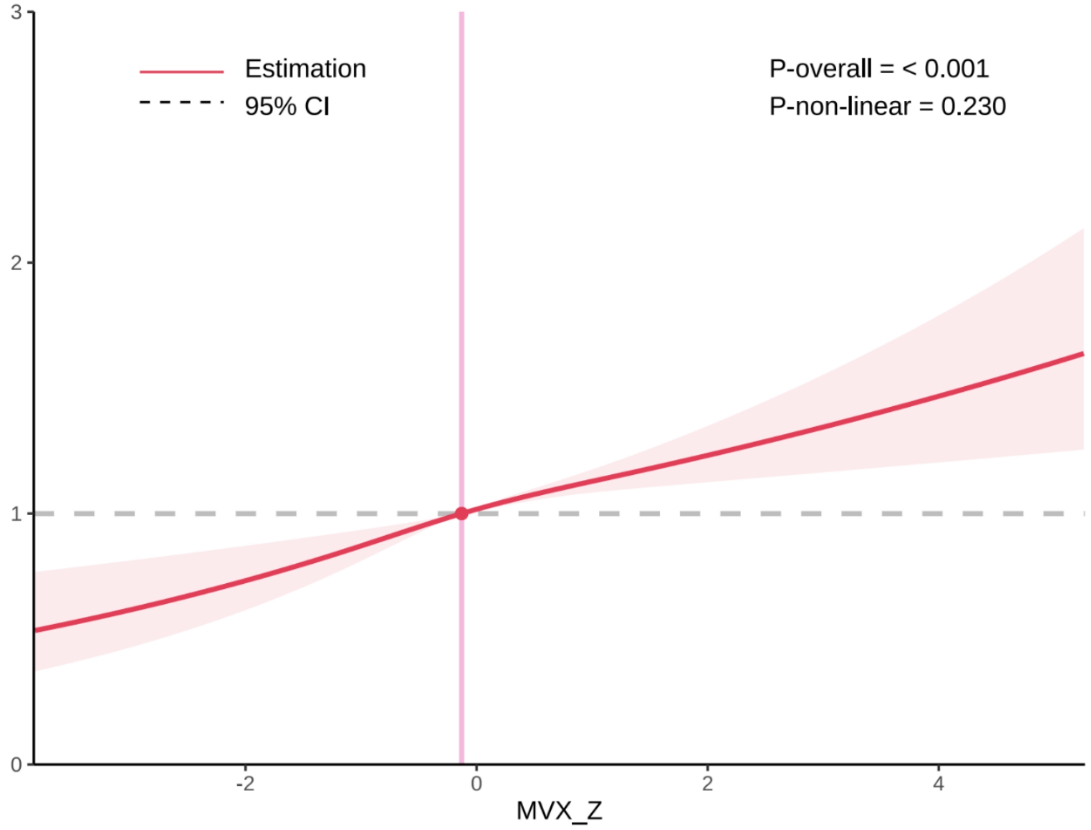

Supplementary Figure 5. Restricted cubic spline curves of the associations between MVX and CVD mortality.

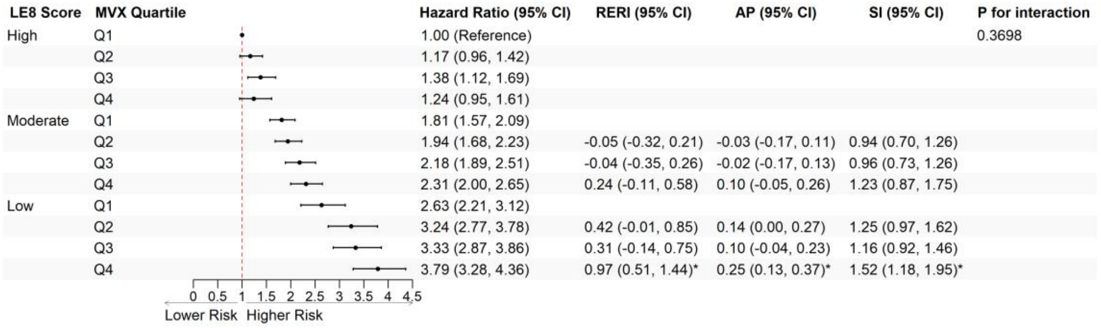

LE8 Score	MVX Quartile	Hazard Ratio (95% CI)	RERI (95% CI)	AP (95% CI)	SI (95% CI)	P for interaction
High	Q1	1.00 (Reference)				0.3698
	Q2	1.17 (0.96, 1.42)				
	Q3	1.38 (1.12, 1.69)				
	Q4	1.24 (0.95, 1.61)				
Moderate	Q1	1.81 (1.57, 2.09)				
	Q2	1.94 (1.68, 2.23)	-0.05 (-0.32, 0.21)	-0.03 (-0.17, 0.11)	0.94 (0.70, 1.26)	
	Q3	2.18 (1.89, 2.51)	-0.04 (-0.35, 0.26)	-0.02 (-0.17, 0.13)	0.96 (0.73, 1.26)	
	Q4	2.31 (2.00, 2.65)	0.24 (-0.11, 0.58)	0.10 (-0.05, 0.26)	1.23 (0.87, 1.75)	
Low	Q1	2.63 (2.21, 3.12)				
	Q2	3.24 (2.77, 3.78)	0.42 (-0.01, 0.85)	0.14 (0.00, 0.27)	1.25 (0.97, 1.62)	
	Q3	3.33 (2.87, 3.86)	0.31 (-0.14, 0.75)	0.10 (-0.04, 0.23)	1.16 (0.92, 1.46)	
	Q4	3.79 (3.28, 4.36)	0.97 (0.51, 1.44)*	0.25 (0.13, 0.37)*	1.52 (1.18, 1.95)*	

Supplementary Figure 6. Combined effects of MVX quartile, LE8, and the risk of myocardial infarction.

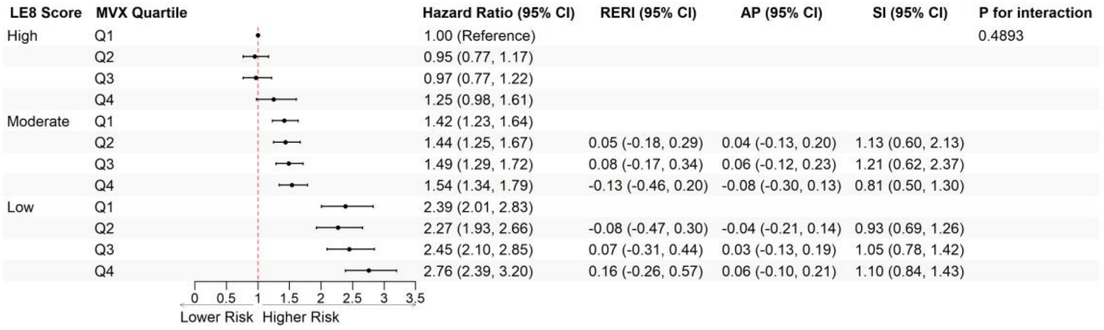

LE8 Score	MVX Quartile	Hazard Ratio (95% CI)	RERI (95% CI)	AP (95% CI)	SI (95% CI)	P for interaction
High	Q1	1.00 (Reference)				0.4893
	Q2	0.95 (0.77, 1.17)				
	Q3	0.97 (0.77, 1.22)				
	Q4	1.25 (0.98, 1.61)				
Moderate	Q1	1.42 (1.23, 1.64)				
	Q2	1.44 (1.25, 1.67)	0.05 (-0.18, 0.29)	0.04 (-0.13, 0.20)	1.13 (0.60, 2.13)	
	Q3	1.49 (1.29, 1.72)	0.08 (-0.17, 0.34)	0.06 (-0.12, 0.23)	1.21 (0.62, 2.37)	
	Q4	1.54 (1.34, 1.79)	-0.13 (-0.46, 0.20)	-0.08 (-0.30, 0.13)	0.81 (0.50, 1.30)	
Low	Q1	2.39 (2.01, 2.83)				
	Q2	2.27 (1.93, 2.66)	-0.08 (-0.47, 0.30)	-0.04 (-0.21, 0.14)	0.93 (0.69, 1.26)	
	Q3	2.45 (2.10, 2.85)	0.07 (-0.31, 0.44)	0.03 (-0.13, 0.19)	1.05 (0.78, 1.42)	
	Q4	2.76 (2.39, 3.20)	0.16 (-0.26, 0.57)	0.06 (-0.10, 0.21)	1.10 (0.84, 1.43)	

Supplementary Figure 7. Combined effects of MVX quartile, LE8, and the risk of heart failure.

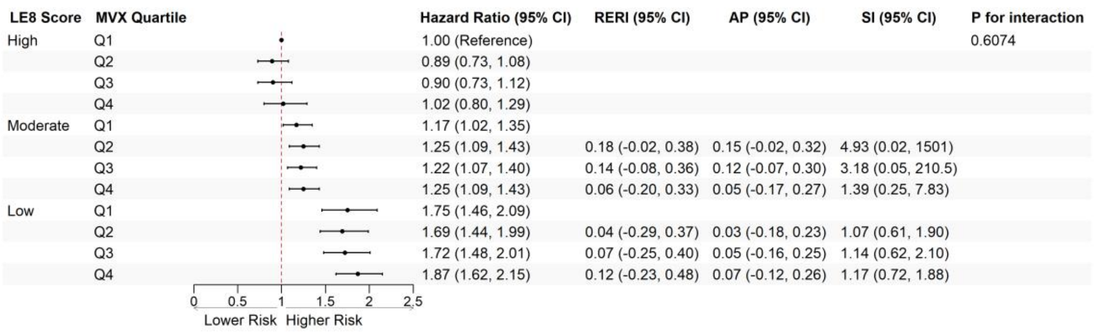

LE8 Score	MVX Quartile	Hazard Ratio (95% CI)	RERI (95% CI)	AP (95% CI)	SI (95% CI)	P for interaction
High	Q1	1.00 (Reference)				0.6074
	Q2	0.89 (0.73, 1.08)				
	Q3	0.90 (0.73, 1.12)				
	Q4	1.02 (0.80, 1.29)				
Moderate	Q1	1.17 (1.02, 1.35)				
	Q2	1.25 (1.09, 1.43)	0.18 (-0.02, 0.38)	0.15 (-0.02, 0.32)	4.93 (0.02, 1501)	
	Q3	1.22 (1.07, 1.40)	0.14 (-0.08, 0.36)	0.12 (-0.07, 0.30)	3.18 (0.05, 210.5)	
	Q4	1.25 (1.09, 1.43)	0.06 (-0.20, 0.33)	0.05 (-0.17, 0.27)	1.39 (0.25, 7.83)	
Low	Q1	1.75 (1.46, 2.09)				
	Q2	1.69 (1.44, 1.99)	0.04 (-0.29, 0.37)	0.03 (-0.18, 0.23)	1.07 (0.61, 1.90)	
	Q3	1.72 (1.48, 2.01)	0.07 (-0.25, 0.40)	0.05 (-0.16, 0.25)	1.14 (0.62, 2.10)	
	Q4	1.87 (1.62, 2.15)	0.12 (-0.23, 0.48)	0.07 (-0.12, 0.26)	1.17 (0.72, 1.88)	

Supplementary Figure 8. Combined effects of MVX quartile, LE8, and the risk of stroke.

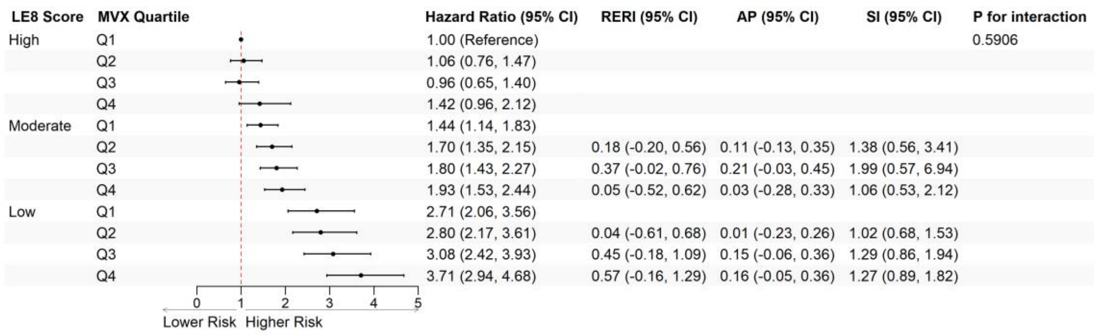

LE8 Score	MVX Quartile	Hazard Ratio (95% CI)	RERI (95% CI)	AP (95% CI)	SI (95% CI)	P for interaction
High	Q1	1.00 (Reference)				0.5906
	Q2	1.06 (0.76, 1.47)				
	Q3	0.96 (0.65, 1.40)				
	Q4	1.42 (0.96, 2.12)				
Moderate	Q1	1.44 (1.14, 1.83)				
	Q2	1.70 (1.35, 2.15)	0.18 (-0.20, 0.56)	0.11 (-0.13, 0.35)	1.38 (0.56, 3.41)	
	Q3	1.80 (1.43, 2.27)	0.37 (-0.02, 0.76)	0.21 (-0.03, 0.45)	1.99 (0.57, 6.94)	
	Q4	1.93 (1.53, 2.44)	0.05 (-0.52, 0.62)	0.03 (-0.28, 0.33)	1.06 (0.53, 2.12)	
Low	Q1	2.71 (2.06, 3.56)				
	Q2	2.80 (2.17, 3.61)	0.04 (-0.61, 0.68)	0.01 (-0.23, 0.26)	1.02 (0.68, 1.53)	
	Q3	3.08 (2.42, 3.93)	0.45 (-0.18, 1.09)	0.15 (-0.06, 0.36)	1.29 (0.86, 1.94)	
	Q4	3.71 (2.94, 4.68)	0.57 (-0.16, 1.29)	0.16 (-0.05, 0.36)	1.27 (0.89, 1.82)	

Supplementary Figure 9. Combined effects of MVX quartile, LE8, and the risk of CVD mortality.

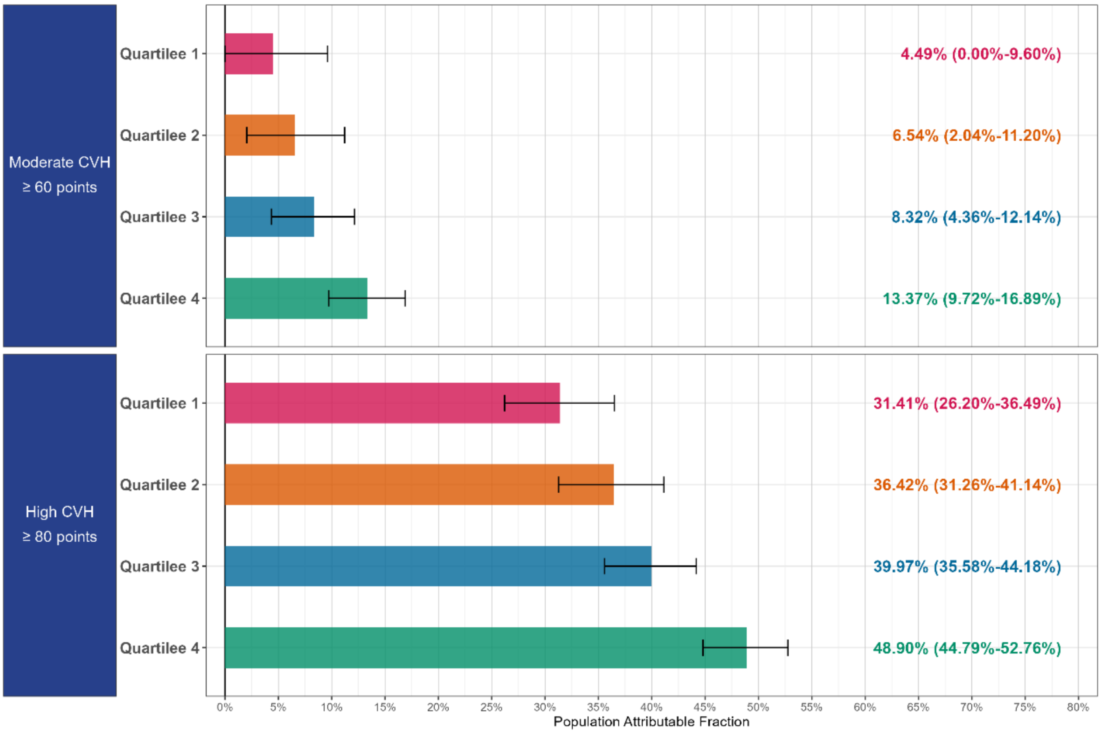

Supplementary Figure 10. The proportion of preventable myocardial infarction cases under counterfactual scenario of cardiovascular health (LE8 scores increased to 60 and 80) within each metabolic vulnerability index quartile. The error bars denote the 95% CI generated by bootstraps.

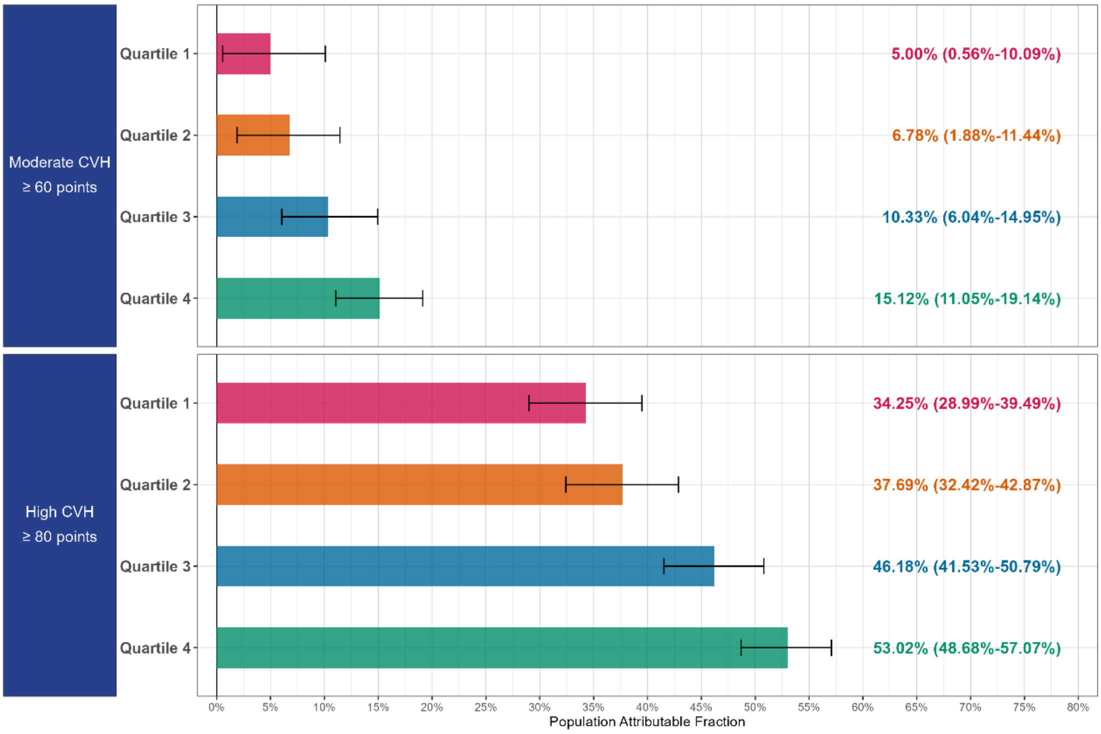

Supplementary Figure 11. The proportion of preventable heart failure cases under counterfactual scenario of cardiovascular health (LE8 scores increased to 60 and 80) within each metabolic vulnerability index quartile. The error bars denote the 95% CI generated by bootstraps.

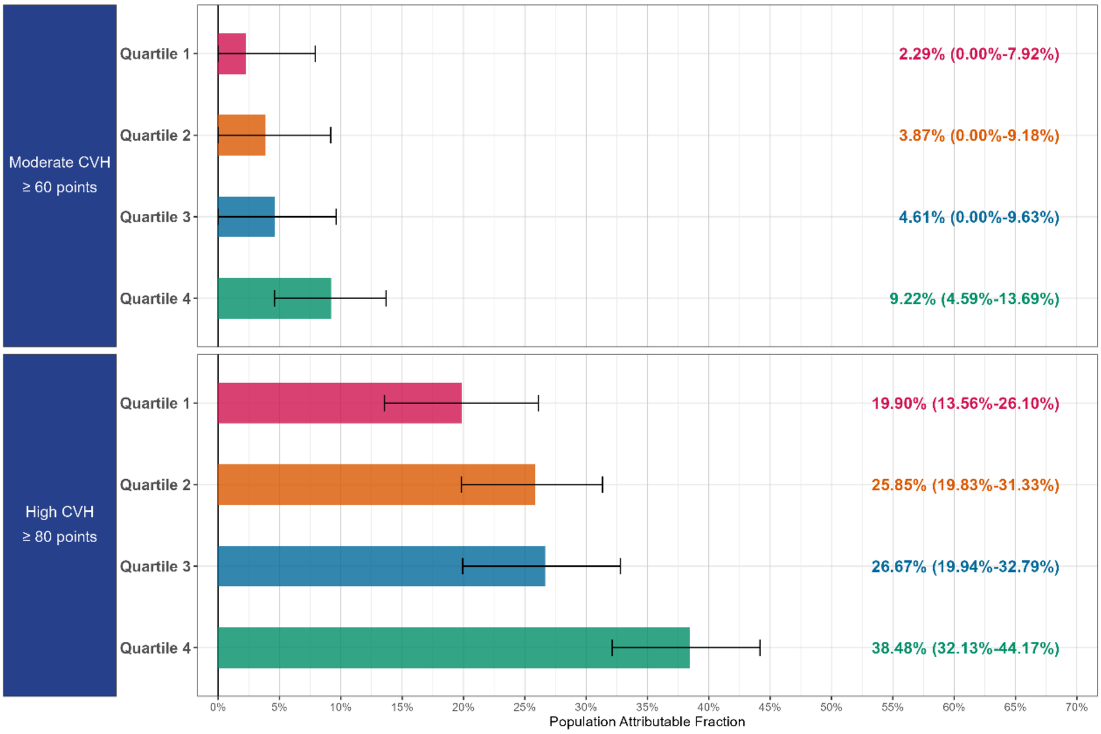

Supplementary Figure 12. The proportion of preventable stroke cases under counterfactual scenario of cardiovascular health (LE8 scores increased to 60 and 80) within each metabolic vulnerability index quartile. The error bars denote the 95% CI generated by bootstraps.

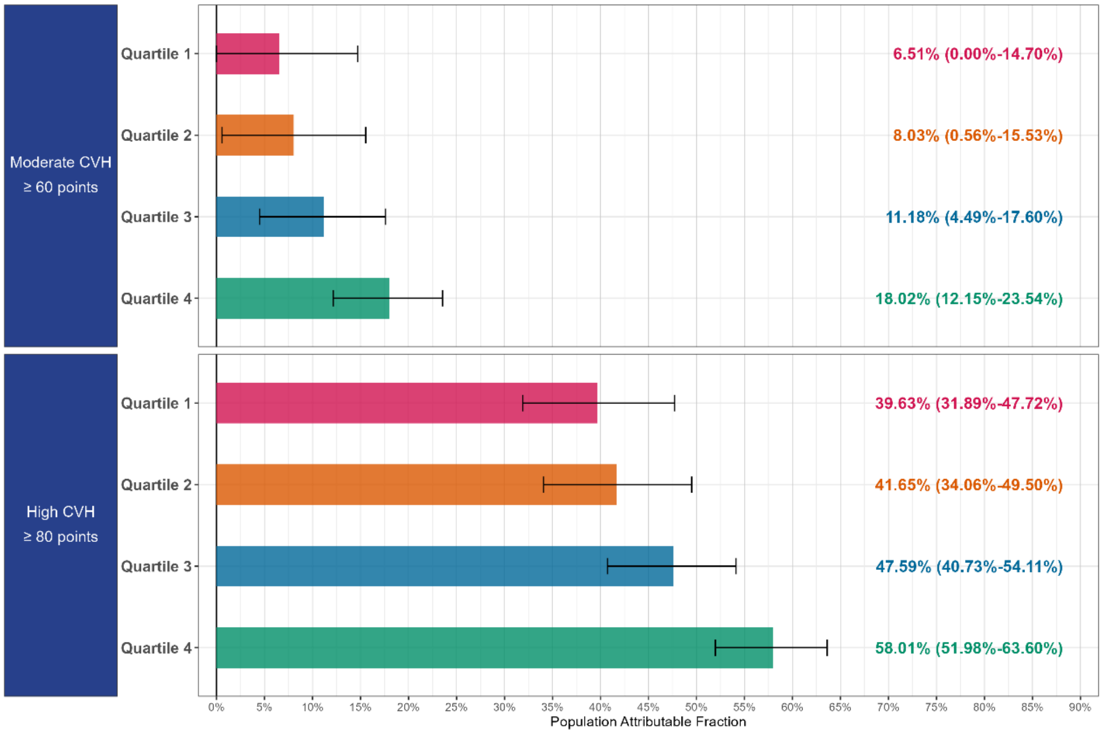

Supplementary Figure 13. The proportion of preventable CVD mortality under counterfactual scenario of cardiovascular health (LE8 scores increased to 60 and 80) within each metabolic vulnerability index quartile. The error bars denote the 95% CI generated by bootstraps.

Subgroup	Category	MVX quartile	Events/n	HR (95% CI)	P-value	P-interaction
Age	≤65 years	Quartil 1	2538/56443	1.00 (Reference)	Reference	<0.001
		Quartil 2	2795/51979	1.08 (1.02, 1.14)	0.005	
		Quartil 3	3235/49829	1.17 (1.11, 1.24)	<0.001	
		Quartil 4	3874/50072	1.26 (1.19, 1.32)	<0.001	
	>65 years	Quartil 1	991/6785	1.00 (Reference)	Reference	
		Quartil 2	1143/7700	1.04 (0.95, 1.13)	0.426	
		Quartil 3	1237/8079	1.05 (0.97, 1.14)	0.251	
		Quartil 4	1333/8248	1.09 (1.00, 1.19)	0.045	
Sex	Female	Quartil 1	1119/33740	1.00 (Reference)	Reference	<0.001
		Quartil 2	1352/32427	1.11 (1.03, 1.20)	0.010	
		Quartil 3	1458/28998	1.22 (1.12, 1.31)	<0.001	
		Quartil 4	2041/29732	1.44 (1.34, 1.55)	<0.001	
	Male	Quartil 1	2410/29488	1.00 (Reference)	Reference	
		Quartil 2	2586/27252	1.06 (1.00, 1.12)	0.037	
		Quartil 3	3014/28910	1.11 (1.06, 1.18)	<0.001	
		Quartil 4	3166/28588	1.11 (1.05, 1.17)	<0.001	
Ethnicity	Other ethnic groups	Quartil 1	171/3214	1.00 (Reference)	Reference	0.401
		Quartil 2	151/2796	0.95 (0.76, 1.19)	0.658	
		Quartil 3	178/2563	1.17 (0.94, 1.44)	0.156	
		Quartil 4	204/2585	1.27 (1.03, 1.56)	0.025	
	White	Quartil 1	3358/60014	1.00 (Reference)	Reference	
		Quartil 2	3787/56883	1.08 (1.03, 1.13)	0.001	
		Quartil 3	4294/55345	1.15 (1.10, 1.20)	<0.001	
		Quartil 4	5003/55735	1.22 (1.17, 1.28)	<0.001	
Binge drinking	No	Quartil 1	3411/61772	1.00 (Reference)	Reference	0.128
		Quartil 2	3805/58305	1.08 (1.03, 1.13)	0.002	
		Quartil 3	4271/56225	1.15 (1.10, 1.20)	<0.001	
		Quartil 4	4909/55854	1.24 (1.18, 1.29)	<0.001	
	Yes	Quartil 1	118/1456	1.00 (Reference)	Reference	
		Quartil 2	133/1374	0.98 (0.76, 1.25)	0.851	
		Quartil 3	201/1683	1.09 (0.86, 1.37)	0.478	
		Quartil 4	298/2466	0.98 (0.78, 1.22)	0.845	
CVH	Poor	Quartil 1	631/5992	1.00 (Reference)	Reference	0.606
		Quartil 2	1016/8834	1.08 (0.98, 1.20)	0.114	
		Quartil 3	1468/11709	1.16 (1.06, 1.28)	0.002	
		Quartil 4	2388/17678	1.27 (1.17, 1.39)	<0.001	
	Intermediate	Quartil 1	2310/37372	1.00 (Reference)	Reference	
		Quartil 2	2510/37452	1.07 (1.01, 1.13)	0.017	
		Quartil 3	2687/36751	1.14 (1.08, 1.21)	<0.001	
		Quartil 4	2610/34708	1.19 (1.12, 1.26)	<0.001	
	Healthy	Quartil 1	588/19864	1.00 (Reference)	Reference	
		Quartil 2	412/13393	1.09 (0.96, 1.23)	0.192	
		Quartil 3	317/9448	1.20 (1.04, 1.37)	0.011	
		Quartil 4	209/5934	1.40 (1.19, 1.64)	<0.001	

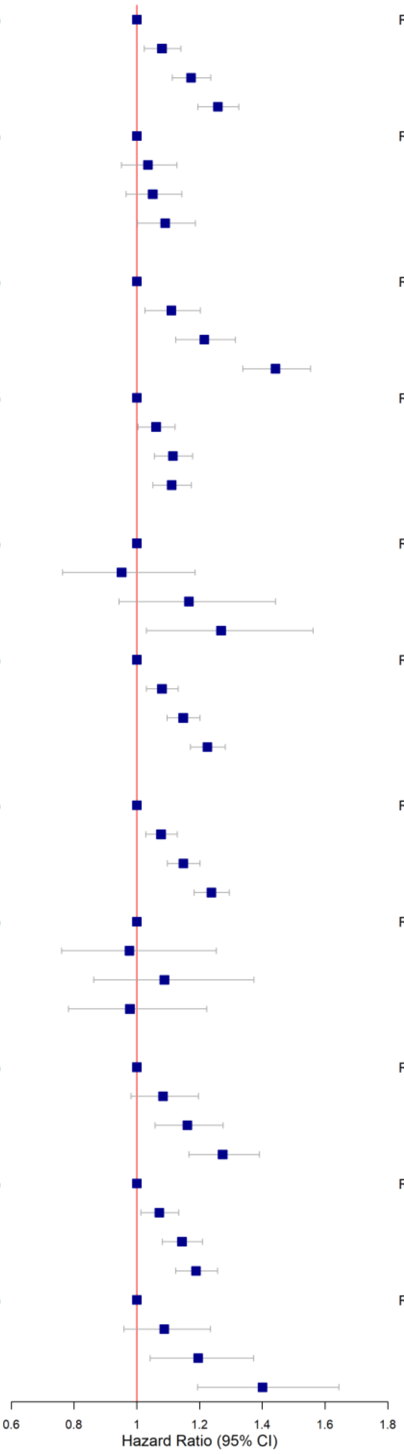

Supplementary Figure 14. Subgroup analyses of the association between MVX quartiles and incident MACE.

Subgroup	Category	MVX quartile	Events/n	HR (95% CI)	P-value	P-interaction
Age	≤65 years	Quartil 1	1191/56443	1.00 (Reference)	Reference	0.026
		Quartil 2	1398/51979	1.14 (1.06, 1.24)	<0.001	
		Quartil 3	1671/49829	1.26 (1.17, 1.36)	<0.001	
		Quartil 4	2040/50072	1.38 (1.29, 1.49)	<0.001	
	>65 years	Quartil 1	416/6785	1.00 (Reference)	Reference	
		Quartil 2	511/7700	1.11 (0.98, 1.27)	0.111	
		Quartil 3	582/8079	1.19 (1.05, 1.35)	0.007	
		Quartil 4	581/8248	1.16 (1.02, 1.32)	0.026	
Sex	Female	Quartil 1	425/33740	1.00 (Reference)	Reference	<0.001
		Quartil 2	549/32427	1.17 (1.03, 1.33)	0.015	
		Quartil 3	584/28998	1.25 (1.11, 1.42)	<0.001	
		Quartil 4	832/29732	1.48 (1.31, 1.67)	<0.001	
	Male	Quartil 1	1182/29488	1.00 (Reference)	Reference	
		Quartil 2	1360/27252	1.13 (1.05, 1.22)	0.002	
		Quartil 3	1669/28910	1.25 (1.16, 1.35)	<0.001	
		Quartil 4	1789/28588	1.27 (1.18, 1.37)	<0.001	
Ethnicity	Other ethnic groups	Quartil 1	86/3214	1.00 (Reference)	Reference	0.502
		Quartil 2	94/2796	1.21 (0.90, 1.62)	0.202	
		Quartil 3	91/2563	1.20 (0.89, 1.61)	0.237	
		Quartil 4	122/2585	1.55 (1.17, 2.05)	0.002	
	White	Quartil 1	1521/60014	1.00 (Reference)	Reference	
		Quartil 2	1815/56883	1.14 (1.06, 1.22)	<0.001	
		Quartil 3	2162/55345	1.25 (1.17, 1.34)	<0.001	
		Quartil 4	2499/55735	1.33 (1.25, 1.42)	<0.001	
Binge drinking	No	Quartil 1	1556/61772	1.00 (Reference)	Reference	0.041
		Quartil 2	1847/58305	1.14 (1.07, 1.22)	<0.001	
		Quartil 3	2165/56225	1.26 (1.18, 1.34)	<0.001	
		Quartil 4	2496/55854	1.36 (1.28, 1.45)	<0.001	
	Yes	Quartil 1	51/1456	1.00 (Reference)	Reference	
		Quartil 2	62/1374	1.06 (0.73, 1.54)	0.767	
		Quartil 3	88/1683	1.07 (0.75, 1.53)	0.699	
		Quartil 4	125/2466	0.93 (0.66, 1.31)	0.682	
CVH	Poor	Quartil 1	295/5992	1.00 (Reference)	Reference	0.513
		Quartil 2	542/8834	1.24 (1.08, 1.43)	0.003	
		Quartil 3	760/11709	1.28 (1.12, 1.47)	<0.001	
		Quartil 4	1231/17678	1.42 (1.25, 1.61)	<0.001	
	Intermediate	Quartil 1	1081/37372	1.00 (Reference)	Reference	
		Quartil 2	1191/37452	1.10 (1.01, 1.19)	0.030	
		Quartil 3	1349/36751	1.23 (1.14, 1.34)	<0.001	
		Quartil 4	1315/34708	1.30 (1.20, 1.41)	<0.001	
	Healthy	Quartil 1	231/19864	1.00 (Reference)	Reference	
		Quartil 2	176/13393	1.22 (1.00, 1.48)	0.051	
		Quartil 3	144/9448	1.44 (1.17, 1.78)	<0.001	
		Quartil 4	75/5934	1.39 (1.07, 1.81)	0.015	

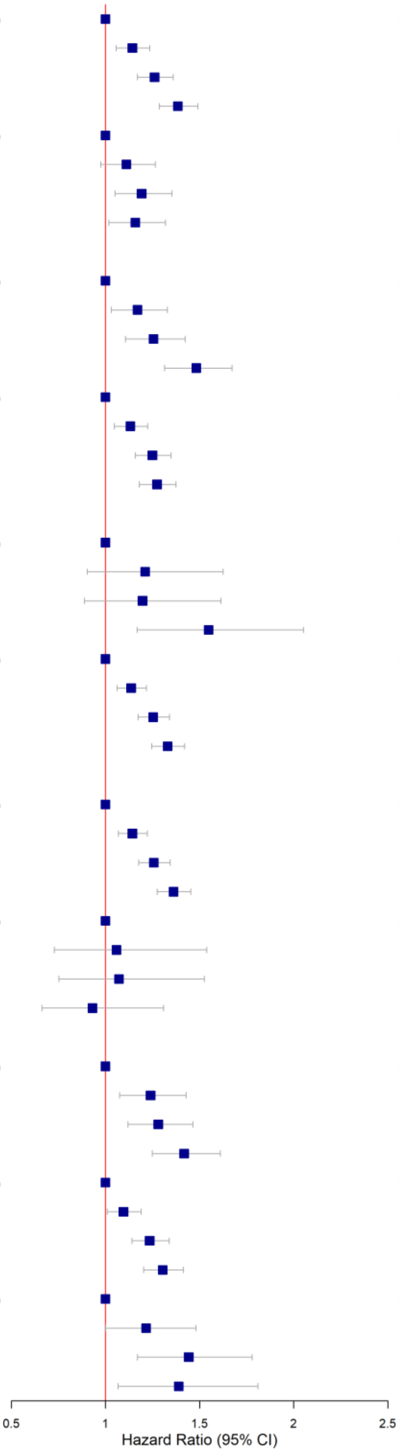

Supplementary Figure 15. Subgroup analyses of the association between MVX quartiles and incident myocardial infarction.

Subgroup	Category	MVX quartile	Events/n	HR (95% CI)	P-value	P-interaction
Age	≤65 years	Quartil 1	997/56443	1.00 (Reference)	Reference	0.036
		Quartil 2	1067/51979	1.02 (0.93, 1.11)	0.694	
		Quartil 3	1262/49829	1.10 (1.02, 1.20)	0.021	
		Quartil 4	1524/50072	1.16 (1.07, 1.26)	<0.001	
	>65 years	Quartil 1	526/6785	1.00 (Reference)	Reference	
		Quartil 2	586/7700	0.99 (0.88, 1.12)	0.883	
		Quartil 3	616/8079	0.96 (0.85, 1.08)	0.490	
		Quartil 4	723/8248	1.06 (0.95, 1.19)	0.308	
Sex	Female	Quartil 1	447/33740	1.00 (Reference)	Reference	<0.001
		Quartil 2	563/32427	1.11 (0.98, 1.26)	0.098	
		Quartil 3	640/28998	1.24 (1.10, 1.40)	<0.001	
		Quartil 4	977/29732	1.53 (1.36, 1.72)	<0.001	
	Male	Quartil 1	1076/29488	1.00 (Reference)	Reference	
		Quartil 2	1090/27252	0.98 (0.90, 1.07)	0.663	
		Quartil 3	1238/28910	0.99 (0.91, 1.08)	0.812	
		Quartil 4	1270/28588	0.95 (0.88, 1.04)	0.261	
Ethnicity	Other ethnic groups	Quartil 1	75/3214	1.00 (Reference)	Reference	0.246
		Quartil 2	51/2796	0.71 (0.50, 1.02)	0.062	
		Quartil 3	69/2563	1.00 (0.72, 1.39)	0.999	
		Quartil 4	75/2585	1.00 (0.72, 1.39)	0.992	
	White	Quartil 1	1448/60014	1.00 (Reference)	Reference	
		Quartil 2	1602/56883	1.03 (0.96, 1.11)	0.383	
		Quartil 3	1809/55345	1.07 (1.00, 1.15)	0.053	
		Quartil 4	2172/55735	1.15 (1.08, 1.23)	<0.001	
Binge drinking	No	Quartil 1	1469/61772	1.00 (Reference)	Reference	0.986
		Quartil 2	1590/58305	1.02 (0.95, 1.09)	0.662	
		Quartil 3	1791/56225	1.07 (1.00, 1.14)	0.068	
		Quartil 4	2093/55854	1.14 (1.07, 1.23)	<0.001	
	Yes	Quartil 1	54/1456	1.00 (Reference)	Reference	
		Quartil 2	63/1374	0.99 (0.69, 1.43)	0.949	
		Quartil 3	87/1683	0.97 (0.69, 1.38)	0.881	
		Quartil 4	154/2466	1.02 (0.74, 1.41)	0.884	
CVH	Poor	Quartil 1	331/5992	1.00 (Reference)	Reference	0.485
		Quartil 2	468/8834	0.95 (0.83, 1.10)	0.493	
		Quartil 3	685/11709	1.03 (0.90, 1.18)	0.652	
		Quartil 4	1104/17678	1.12 (0.99, 1.27)	0.068	
	Intermediate	Quartil 1	970/37372	1.00 (Reference)	Reference	
		Quartil 2	1036/37452	1.04 (0.96, 1.14)	0.330	
		Quartil 3	1084/36751	1.08 (0.99, 1.18)	0.067	
		Quartil 4	1057/34708	1.12 (1.03, 1.23)	0.010	
	Healthy	Quartil 1	222/19864	1.00 (Reference)	Reference	
		Quartil 2	149/13393	1.03 (0.84, 1.27)	0.765	
		Quartil 3	109/9448	1.07 (0.85, 1.34)	0.583	
		Quartil 4	86/5934	1.51 (1.17, 1.95)	0.001	

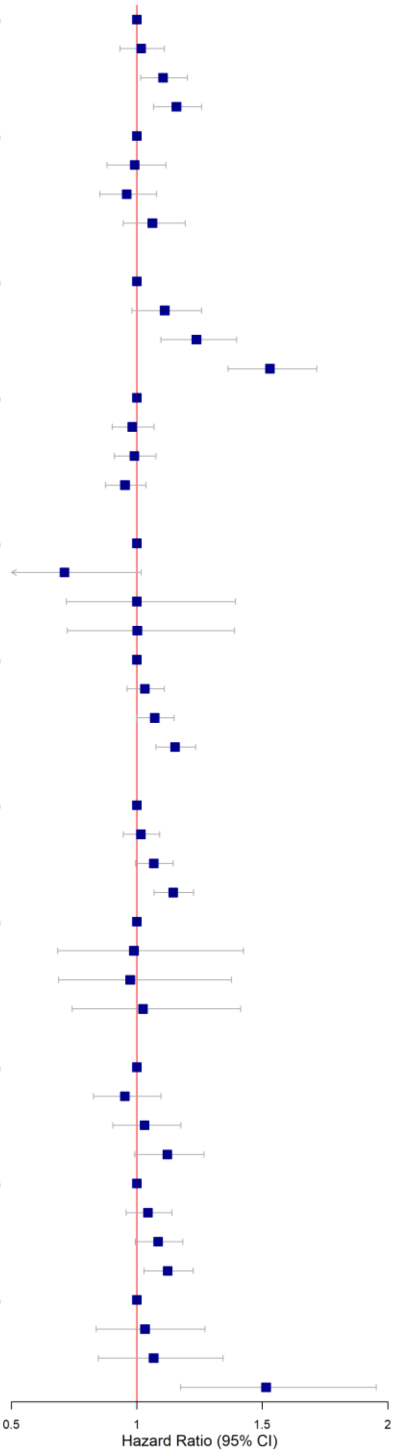

Supplementary Figure 16. Subgroup analyses of the association between MVX quartiles and incident heart failure.

Subgroup	Category	MVX quartile	Events/n	HR (95% CI)	P-value	P-interaction
Age	≤65 years	Quartil 1	984/56443	1.00 (Reference)	Reference	0.970
		Quartil 2	1025/51979	1.04 (0.95, 1.14)	0.365	
		Quartil 3	1055/49829	1.03 (0.94, 1.12)	0.574	
		Quartil 4	1203/50072	1.06 (0.97, 1.16)	0.173	
	>65 years	Quartil 1	370/6785	1.00 (Reference)	Reference	
		Quartil 2	427/7700	1.03 (0.90, 1.19)	0.670	
		Quartil 3	458/8079	1.04 (0.91, 1.20)	0.567	
		Quartil 4	495/8248	1.09 (0.95, 1.25)	0.229	
Sex	Female	Quartil 1	501/33740	1.00 (Reference)	Reference	<0.001
		Quartil 2	560/32427	1.04 (0.92, 1.18)	0.487	
		Quartil 3	583/28998	1.12 (0.99, 1.26)	0.072	
		Quartil 4	767/29732	1.28 (1.14, 1.44)	<0.001	
	Male	Quartil 1	853/29488	1.00 (Reference)	Reference	
		Quartil 2	892/27252	1.05 (0.96, 1.16)	0.277	
		Quartil 3	930/28910	1.00 (0.91, 1.10)	0.984	
		Quartil 4	931/28588	0.96 (0.87, 1.06)	0.459	
Ethnicity	Other ethnic groups	Quartil 1	74/3214	1.00 (Reference)	Reference	0.027
		Quartil 2	44/2796	0.64 (0.44, 0.94)	0.021	
		Quartil 3	67/2563	1.01 (0.72, 1.42)	0.944	
		Quartil 4	56/2585	0.79 (0.56, 1.13)	0.207	
	White	Quartil 1	1280/60014	1.00 (Reference)	Reference	
		Quartil 2	1408/56883	1.07 (0.99, 1.15)	0.093	
		Quartil 3	1446/55345	1.05 (0.97, 1.13)	0.255	
		Quartil 4	1642/55735	1.10 (1.02, 1.19)	0.011	
Binge drinking	No	Quartil 1	1303/61772	1.00 (Reference)	Reference	0.479
		Quartil 2	1399/58305	1.05 (0.97, 1.13)	0.230	
		Quartil 3	1444/56225	1.04 (0.97, 1.12)	0.277	
		Quartil 4	1600/55854	1.10 (1.02, 1.18)	0.017	
	Yes	Quartil 1	51/1456	1.00 (Reference)	Reference	
		Quartil 2	53/1374	0.96 (0.65, 1.41)	0.833	
		Quartil 3	69/1683	0.95 (0.66, 1.38)	0.796	
		Quartil 4	98/2466	0.85 (0.59, 1.22)	0.375	
CVH	Poor	Quartil 1	231/5992	1.00 (Reference)	Reference	0.684
		Quartil 2	332/8834	0.97 (0.82, 1.15)	0.727	
		Quartil 3	456/11709	0.99 (0.85, 1.16)	0.911	
		Quartil 4	717/17678	1.05 (0.90, 1.21)	0.553	
	Intermediate	Quartil 1	849/37372	1.00 (Reference)	Reference	
		Quartil 2	949/37452	1.09 (1.00, 1.20)	0.055	
		Quartil 3	933/36751	1.08 (0.98, 1.18)	0.117	
		Quartil 4	894/34708	1.10 (1.00, 1.21)	0.053	
	Healthy	Quartil 1	274/19864	1.00 (Reference)	Reference	
		Quartil 2	171/13393	0.95 (0.78, 1.15)	0.575	
		Quartil 3	124/9448	0.97 (0.78, 1.20)	0.761	
		Quartil 4	87/5934	1.17 (0.92, 1.50)	0.210	

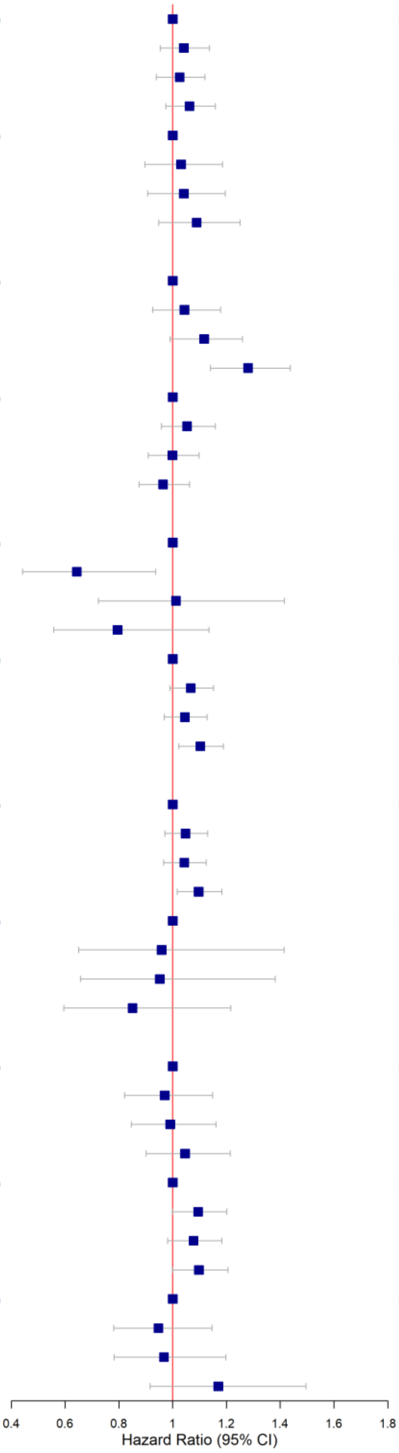

Supplementary Figure 17. Subgroup analyses of the association between MVX quartiles and incident stroke.

Subgroup	Category	MVX quartile	Events/n	HR (95% CI)	P-value	P-interaction
Age	≤65 years	Quartil 1	371/56443	1.00 (Reference)	Reference	0.056
		Quartil 2	470/51979	1.19 (1.04, 1.36)	0.013	
		Quartil 3	572/49829	1.30 (1.14, 1.49)	<0.001	
		Quartil 4	732/50072	1.43 (1.25, 1.62)	<0.001	
	>65 years	Quartil 1	219/6785	1.00 (Reference)	Reference	
		Quartil 2	254/7700	1.05 (0.88, 1.26)	0.587	
		Quartil 3	274/8079	1.05 (0.88, 1.26)	0.593	
		Quartil 4	333/8248	1.24 (1.04, 1.47)	0.018	
Sex	Female	Quartil 1	155/33740	1.00 (Reference)	Reference	0.004
		Quartil 2	203/32427	1.17 (0.95, 1.44)	0.142	
		Quartil 3	239/28998	1.36 (1.11, 1.67)	0.003	
		Quartil 4	368/29732	1.72 (1.41, 2.08)	<0.001	
	Male	Quartil 1	435/29488	1.00 (Reference)	Reference	
		Quartil 2	521/27252	1.14 (1.01, 1.30)	0.042	
		Quartil 3	607/28910	1.17 (1.03, 1.33)	0.014	
		Quartil 4	697/28588	1.25 (1.10, 1.41)	<0.001	
Ethnicity	Other ethnic groups	Quartil 1	21/3214	1.00 (Reference)	Reference	0.397
		Quartil 2	23/2796	1.17 (0.65, 2.12)	0.607	
		Quartil 3	34/2563	1.66 (0.95, 2.88)	0.072	
		Quartil 4	36/2585	1.68 (0.97, 2.92)	0.063	
	White	Quartil 1	569/60014	1.00 (Reference)	Reference	
		Quartil 2	701/56883	1.14 (1.02, 1.28)	0.018	
		Quartil 3	812/55345	1.20 (1.08, 1.34)	<0.001	
		Quartil 4	1029/55735	1.36 (1.23, 1.52)	<0.001	
Binge drinking	No	Quartil 1	570/61772	1.00 (Reference)	Reference	0.160
		Quartil 2	690/58305	1.13 (1.01, 1.27)	0.028	
		Quartil 3	793/56225	1.20 (1.08, 1.34)	0.001	
		Quartil 4	994/55854	1.38 (1.25, 1.54)	<0.001	
	Yes	Quartil 1	20/1456	1.00 (Reference)	Reference	
		Quartil 2	34/1374	1.47 (0.84, 2.57)	0.173	
		Quartil 3	53/1683	1.60 (0.95, 2.70)	0.079	
		Quartil 4	71/2466	1.28 (0.77, 2.14)	0.346	
CVH	Poor	Quartil 1	140/5992	1.00 (Reference)	Reference	0.642
		Quartil 2	215/8834	1.04 (0.84, 1.28)	0.745	
		Quartil 3	325/11709	1.14 (0.94, 1.40)	0.183	
		Quartil 4	553/17678	1.33 (1.10, 1.60)	0.003	
	Intermediate	Quartil 1	365/37372	1.00 (Reference)	Reference	
		Quartil 2	448/37452	1.20 (1.05, 1.38)	0.008	
		Quartil 3	482/36751	1.28 (1.12, 1.47)	<0.001	
		Quartil 4	478/34708	1.37 (1.19, 1.56)	<0.001	
	Healthy	Quartil 1	85/19864	1.00 (Reference)	Reference	
		Quartil 2	61/13393	1.08 (0.78, 1.50)	0.650	
		Quartil 3	39/9448	0.97 (0.66, 1.42)	0.870	
		Quartil 4	34/5934	1.52 (1.01, 2.28)	0.045	

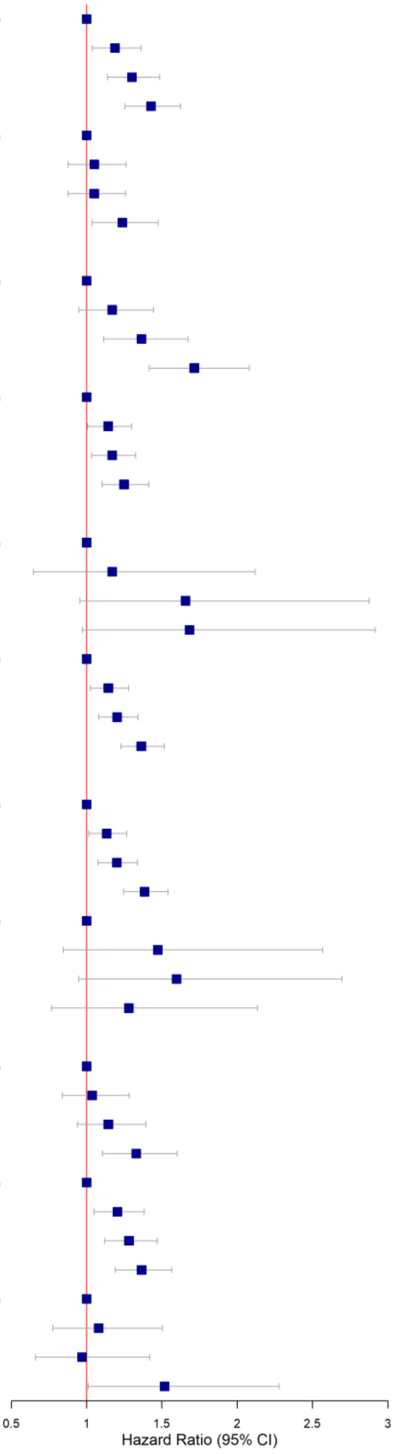

Supplementary Figure 18. Subgroup analyses of the association between MVX quartiles and cardiovascular mortality.

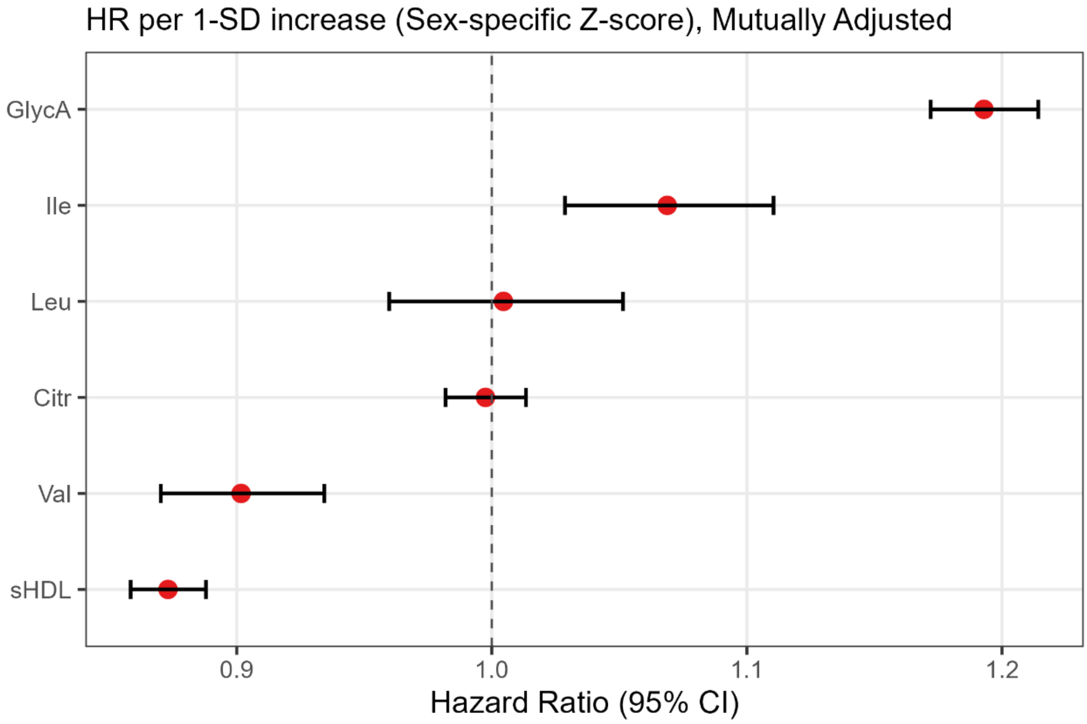

Supplementary Figure 19. Hazard ratios for incident MACE per 1-SD increase in sex-specific standardized MVX biomarkers.

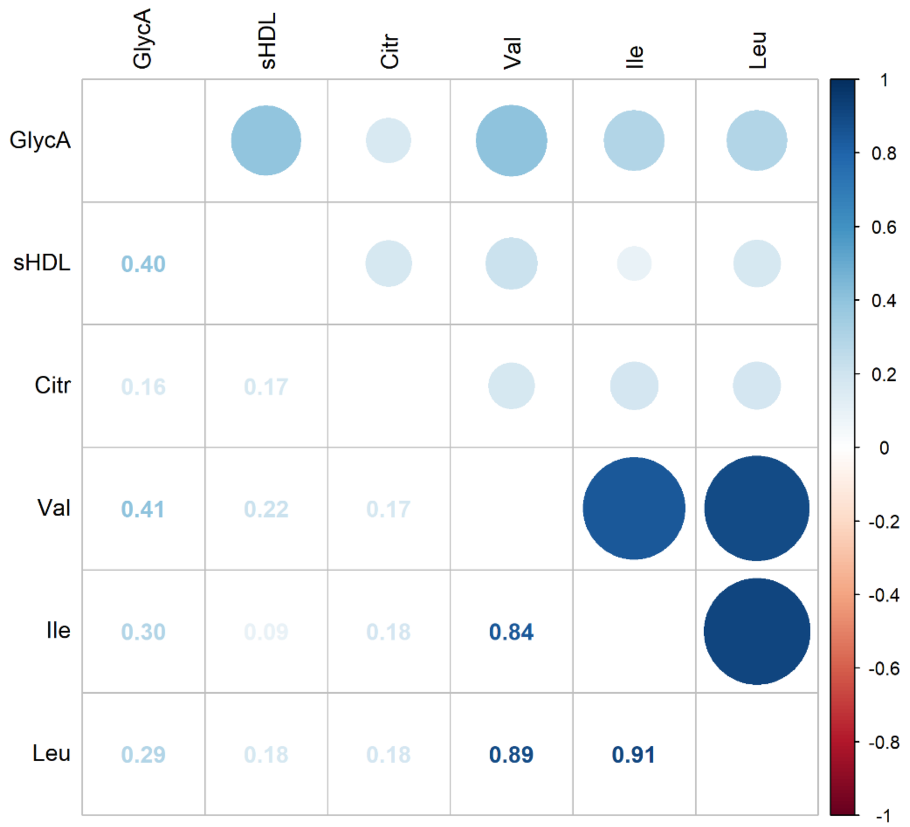

Supplementary Figure 20. Correlations among the six biomarkers comprising the MVX score.

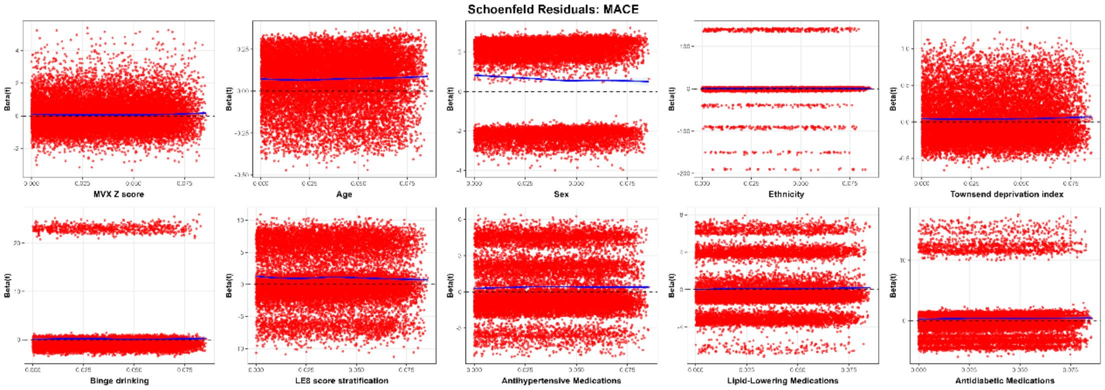

Supplementary Figure 21. Assessment of the proportional hazards assumption for the MACE.

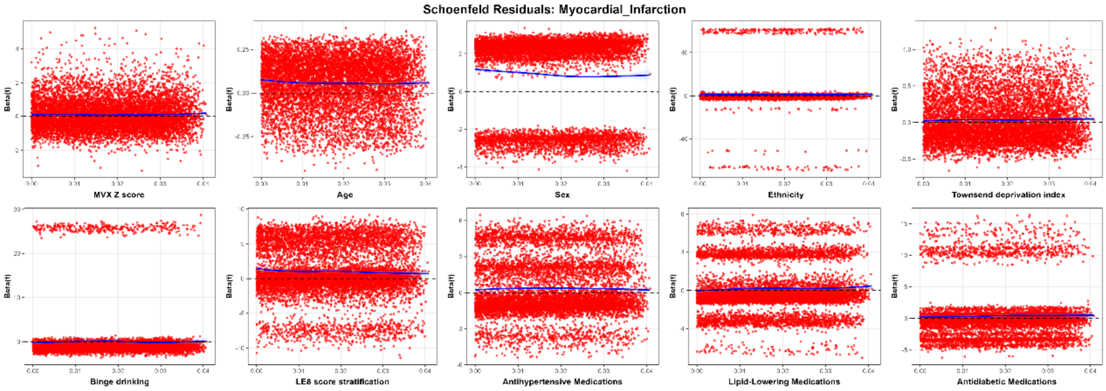

Supplementary Figure 22. Assessment of the proportional hazards assumption for the myocardial infarction.

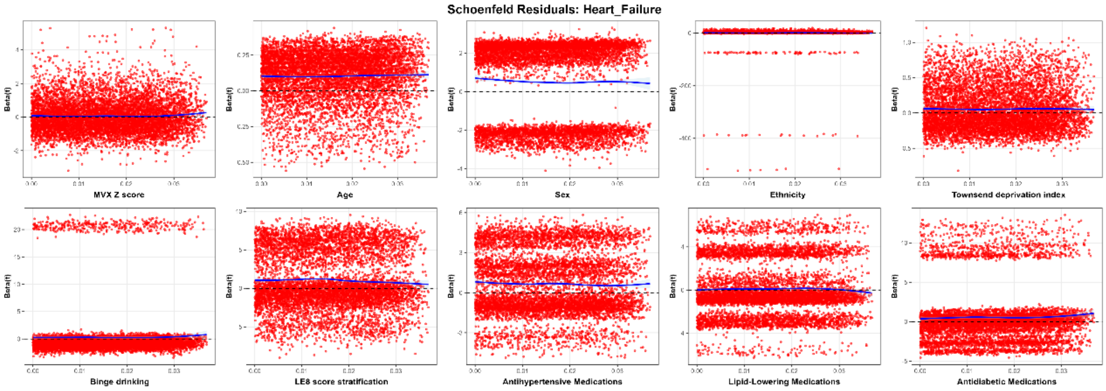

Supplementary Figure 23. Assessment of the proportional hazards assumption for the heart failure.

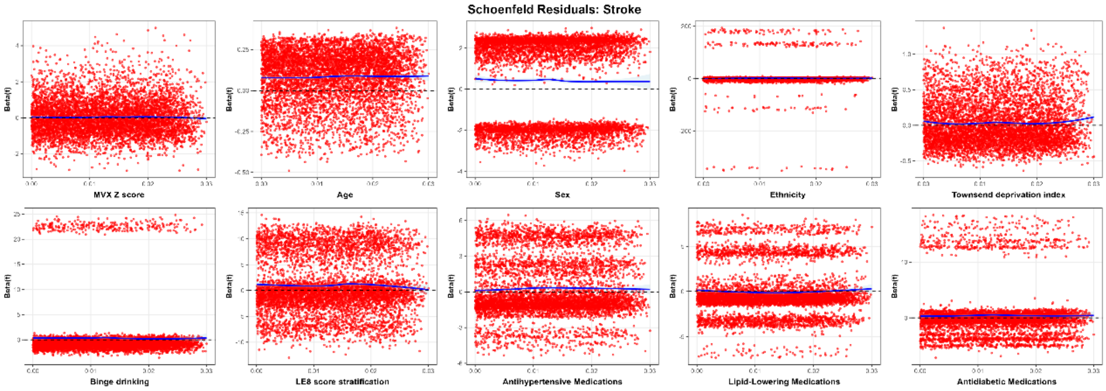

Supplementary Figure 24. Assessment of the proportional hazards assumption for the stroke.

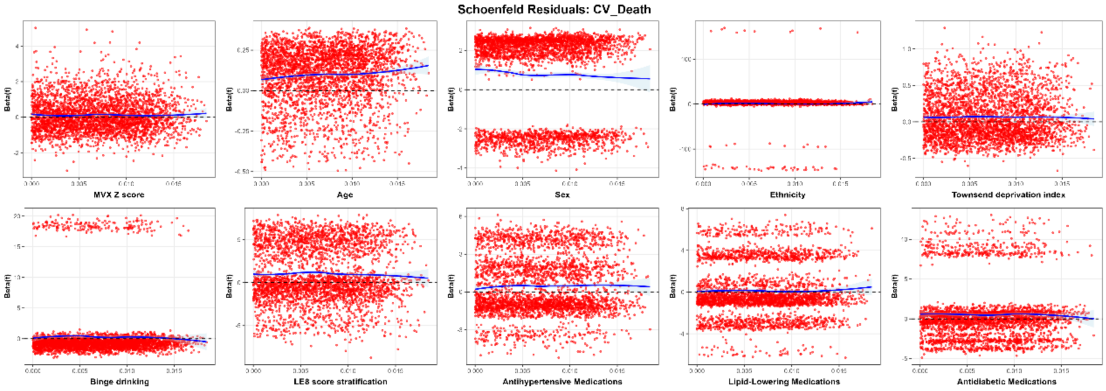

Supplementary Figure 25. Assessment of the proportional hazards assumption for the CVD mortality.
